# Nanobody inhibitors of Plexin-B1 identify allostery in plexin–semaphorin interactions and signaling

**DOI:** 10.1016/j.jbc.2023.104740

**Published:** 2023-04-23

**Authors:** Richard Cowan, Martina Trokter, Arkadiusz Oleksy, Marina Fedorova, Kovilen Sawmynaden, Thomas Worzfeld, Stefan Offermanns, David Matthews, Mark D. Carr, Gareth Hall

**Affiliations:** 1 Department of Molecular and Cell Biology, Leicester Institute of Structural and Chemical Biology, University of Leicester, Leicester, UK; 2 LifeArc, Centre for Therapeutics Discovery, Stevenage, UK; 3 Institute of Pharmacology, University of Marburg, Marburg, Germany; 4 Department of Pharmacology, Max-Planck Institute for Heart and Lung Research, Bad Nauheim, Germany

**Keywords:** single-domain antibody (sdAb, nanobody, VHH), cancer, phage display, multiple sclerosis, semaphorin

## Abstract

Plexin-B1 is a receptor for the cell surface semaphorin, Sema4D. This signaling system has been implicated in a variety of human diseases, including cancer, multiple sclerosis and osteoporosis. While inhibitors of the Plexin-B1:Sema4D interaction have been previously reported, understanding their mechanism has been hindered by an incomplete structural view of Plexin-B1. In this study, we have raised and characterized a pair of nanobodies that are specific for mouse Plexin-B1 and which inhibit the binding of Sema4D to mouse Plexin-B1 and its biological activity. Structural studies of these nanobodies reveal that they inhibit the binding of Sema4D in an allosteric manner, binding to epitopes not previously reported. In addition, we report the first unbound structure of human Plexin-B1, which reveals that Plexin-B1 undergoes a conformational change on Sema4D binding. These changes mirror those seen upon binding of allosteric peptide modulators, which suggests a new model for understanding Plexin-B1 signaling and provides a potential innovative route for therapeutic modulation of Plexin-B1.

The plexins are a family of cell surface single transmembrane domain receptors for cell surface and soluble semaphorins. The semaphorin–plexin signaling system has been widely studied due to its roles in a diverse set of activities, such as neuronal connectivity and growth, cancers, cell migration, and immune responses. The semaphorin–plexin system is found in both vertebrates and invertebrates, as well as in simpler single and multicellular organisms (1).

Plexin-B1 interacts with either Sema4A (2), Sema4B (3), Sema4D (4), or Sema3C (3) and has been found to be involved in angiogenesis and axonal guidance (5), as well as the proliferation and migration of neuronal, epithelial, and tumor cells (6, 7, 8). Bone remodeling has been found to involve plexin-B:sema4 signaling, specifically, osteoclast-expressed Sema4D suppresses bone formation (9). A model system for multiple sclerosis, experimental autoimmune encephalomyelitis, has also been shown to involve the Plexin-B1:Sema4D interaction in its pathogenesis (10).

The intracellular region of Plexin-B1 interacts directly with some members of the Rho family of small GTPases through the Rho GTPase binding domain (RBD), which splits the GTPase-activating protein (GAP) domain of Plexin-B1 into two segments. The Plexin-B1 GAP domain binds R-Ras but does not bind H-Ras (11). The cytoplasmic domain of plexin-B proteins also includes a PDZ-domain binding peptide, which interacts with either LARG or PDZ-RhoGEF (12, 13). Although initial structural studies of the GAP domain of Plexin-B1 suggested that it was dimeric in the resting state (14), later studies of the full-length intracellular domain showed that a short region C terminal of the RBD, termed the coupling loop, binds to the RBD and the N-terminal GAP domain, preventing dimerization (11). These multiple interactions of the intracellular domain of Plexin-B1 support its role in several distinct cellular responses through the presence or absence of different cellular effectors.

The extracellular domains of plexins consist of three domain types: the IPT domain (immunoglobulin domain shared by plexins and transcription factors), the PSI domain (lexins, semaphorins, and integrins), and the sema domain. The PSI domains are small, disulfide-rich adapter domains, typically found between some of the immunoglobulin-like IPT domains, as well as between the sema domain and the first IPT domain. The sema domain is a seven-bladed β-propeller domain (Fig. 1*A*), with a large insert which gives the sema domain a distinctive, asymmetrical shape (15). Sema domains are also found in the related MET and RON receptor tyrosine kinases, which can serve as a coreceptors for plexin signaling (1). Semaphorins are similar to plexins but lack IPT domains. Some semaphorins also contain thrombospondin domains in their extracellular region. The sema domain forms the ligand-binding domain of the semaphorins and plexins, mediating their interactions, as well as interactions with coreceptors (16) or autoinhibitory interactions (17).

**Figure 1:**
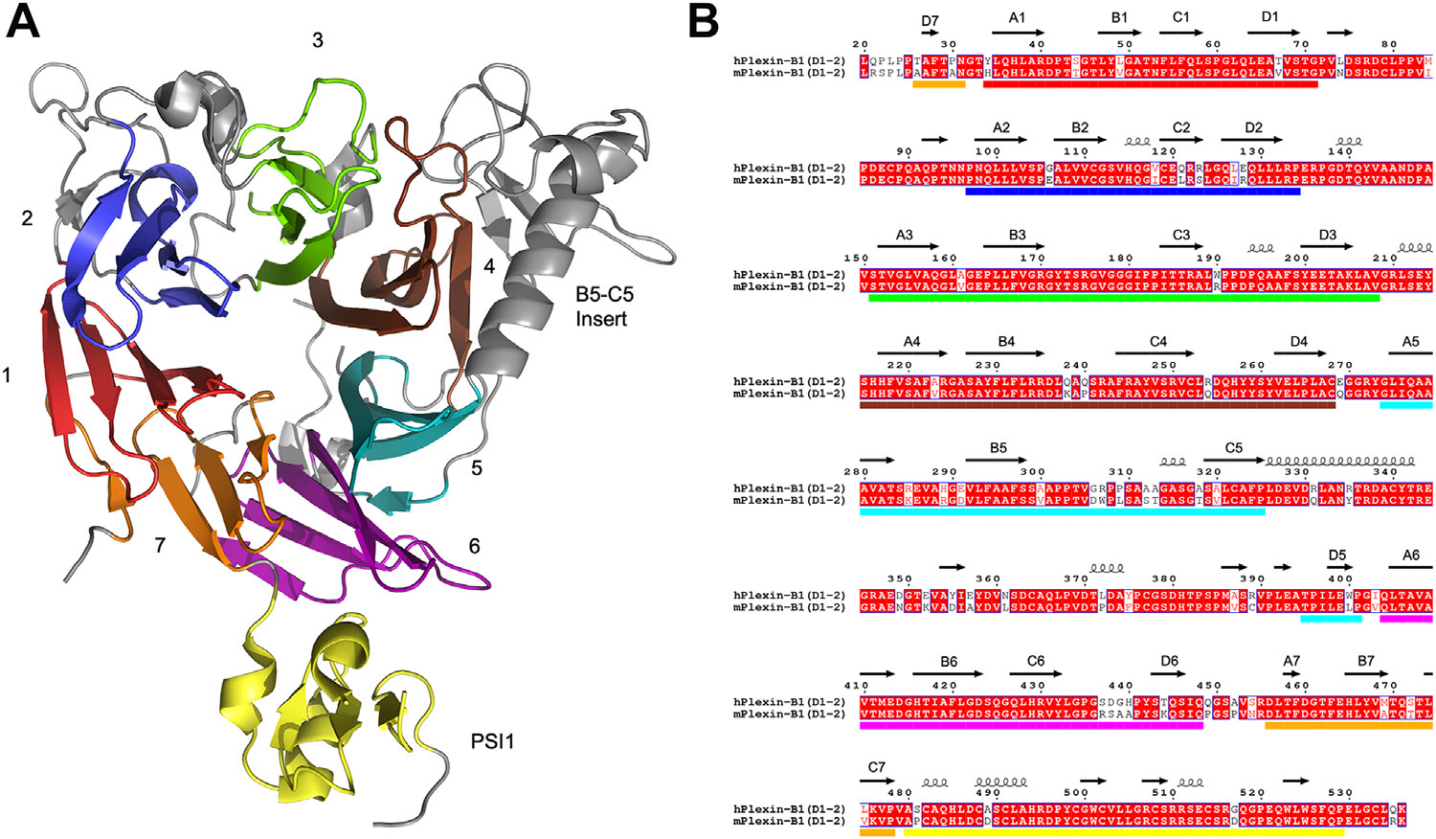
**Architecture of the Plexin-B1 semaphorin and PSI1 domains.***A*, the structure of the Sema4D-bound human Plexin-B1 sema and PSI1 domains (PDB:3OL2, (18)) shows the conserved, seven-bladed B- β-propellor architecture of the sema domain and compact form of the PSI domain. The individual blades of the β-propellor are colored as follows: 1—*red*, *2*—*blue*, *3*—*green*, 4—*brown*, 5—*cyan*, 6—*magenta*, and 7—*orange*. The PSI domain is colored *yellow*. The large inserts of the sema domain are *not colored*. *B*, sequence alignment of the human and mouse Plexin-B1 sema and PSI1 regions showing the high sequence identity between the two species. Secondary structure indicated above the sequences is derived from pdb:3OL2, and the *colored boxes* below the sequences indicate the sema domain blades, and PSI domain, with color scheme as (*A*) The β-strand IDs of the sema domain are indicated above the sequence. The figure was prepared using Pymol (The PyMOL Molecular Graphics System, Version 2.3.2 Schrödinger, LLC) and ESPript 3.0 (45).

The Plexin-B1 extracellular region contains 11 separate domains, with a N-terminal sema domain, followed by a PSI (PSI1), an IPT domain (IPT1), and a second PSI domain (PSI2). Following the second PSI domain is a large, 230 amino acid mucin-like domain, which is unique to the plexin-B family. Following the mucin-like domain are a further IPT domain (IPT2), PSI domain (PSI3), and a final four IPT domains (IPT3-6) (18). The overall architecture of the plexin-A and plexin-B extracellular regions are conserved, with the exception of the insertion of the mucin-like domain into the plexin-Bs (18). A feature of Plexin-B1, also shared with Plexin-B2, is the presence of a proprotein convertase cleavage site in the IPT5 domain. Removal of this site results in significantly reduced Sema4D binding to Plexin-B1-expressing cells but does not affect the surface expression levels of Plexin-B1 (19). Human and murine Plexin-B1 are highly conserved with a sequence identity of 87.3% for the full-length proteins and 87.6% sequence identity for the sema and PSI1 domains (Fig. 1*B*).

The extracellular domains of plexins have undergone significant structural study, although the plexin-As have been more extensively studied than the plexin Bs, C, or D, and have thus served as a model for the extracellular region of the whole plexin superfamily. The ten domain extracellular region of Plexin-A1 in the apo state forms a ring-like autoinhibited conformation in both crystal structures and negative stain electron microscopy (17).

The interaction of the sema domains of plexins and semaphorins has also been structurally studied, and this identified that the interaction between the sema domains occurs on the top face of the domain. No extensive structural changes occur in the Plexin-A2:Sema6A complex between the free and the bound states of both Plexin-A2 and Sema6A (18), nor is there any change in the conformation of Sema4D between the apo and Plexin-B1 bound states (18, 20). From these studies, it has been concluded that there are no changes in the conformation of the sema domain of Plexin-B1 on semaphorin binding and that the autoinhibited conformation of the extracellular domain is disrupted by interdomain movements in Plexin-B1, driven by the dimeric state of Sema4D, similar to Plexin-A1 (17). A peptide-based macrocycle inhibitor of the Plexin-B1:Sema4D interaction has been reported, called PB1m6, which binds to an exposed groove in the Plexin-B1 sema domain and inhibits Sema4D binding through an unknown mechanism which reduces the affinity of Plexin-B1 for Sema4D (21). This peptide has been further developed by dimerizing through a PEG-based linker (22) or grafting into loops in a human Fc fragment (23). An additional peptide has also been shown to bind to Plexin-B1, and when grafted into Fc fragments, to act as an agonist, noncompetitively with Sema4D binding (23).

Therapeutic targeting of the plexin-B:semaphorin interaction has been suggested as a mechanism for the treatment of several conditions, including cancer, multiple sclerosis, osteoporosis (24, 25) and diabetic retinopathy (26). A monoclonal antibody targeted on Sema4D, VX15/2503 (Pepinemab), has completed phase 2 trials for the treatment of non-small-cell lung carcinoma in combination with avelumab (Clinicaltrials.gov identifier: NCT03268057). In addition, antibodies directed against Plexin-B1 which block the interaction of human Plexin-B1 with ErbB-2 have been developed, although these do not affect Sema4D binding (27). Generation and characterization of additional inhibitors of the Plexin-B1:Sema4D interaction would potentially allow the treatment of further conditions which involve Plexin-B1:Sema4D signaling and give improved specificity and properties over the available inhibitors (21, 27).

In this communication, we report the structure of two different nanobodies [antigen binding fragment of heavy chain only antibodies (VHHs)] in complex with the sema and PSI1 domains of mouse Plexin-B1, both of which appear to inhibit the binding of Sema4D to Plexin-B1 in an allosteric manner. The nanobodies bind to a conformation of the sema domain which is similar to the Sema4D bound (18) and macrocycle or peptide-bound conformation (21, 23). These nanobodies bind to sites that are both distinct from each other and to the already described macrocyclic peptides, demonstrating the existence of multiple allosteric regulatory sites on the sema domain of Plexin-B1. In addition, we report the first apo structure of the sema and PSI1 domains of human Plexin-B1, which reveals that the sema domain of Plexin-B1 undergoes a significant conformational change upon either Sema4D or allosteric peptide binding. This discovery potentially changes the existing understanding of the mechanisms of plexin-B signaling and points to several new opportunities to understand plexin-B signaling and its interactions with coreceptors, as well as demonstrating that there are multiple possibilities for therapeutic modulation of the plexin-B:semaphorin interaction.

## Results

### Identification and characterization of anti-Plexin-B1 nanobodies

To generate VHHs that bind Plexin-B1 and inhibit Sema4D binding, a huarizo was immunized with mouse Plexin-B1 domains 1 and 2 (D1-2, 20–535). A VHH phage display library was then generated from isolated lymphocyte mRNA. The library contained 3 × 10^9^ clones, and ∼80% of the clones encoded a VHH, based on the results of colony PCR and sequencing of 96 random clones. Sequencing results also revealed a reasonable diversity of complementarity-determining region 3 (CDR3) sequences. Three rounds of phage display selection were carried out to enrich for Plexin-B1-specific VHHs.

**Figure 2:**
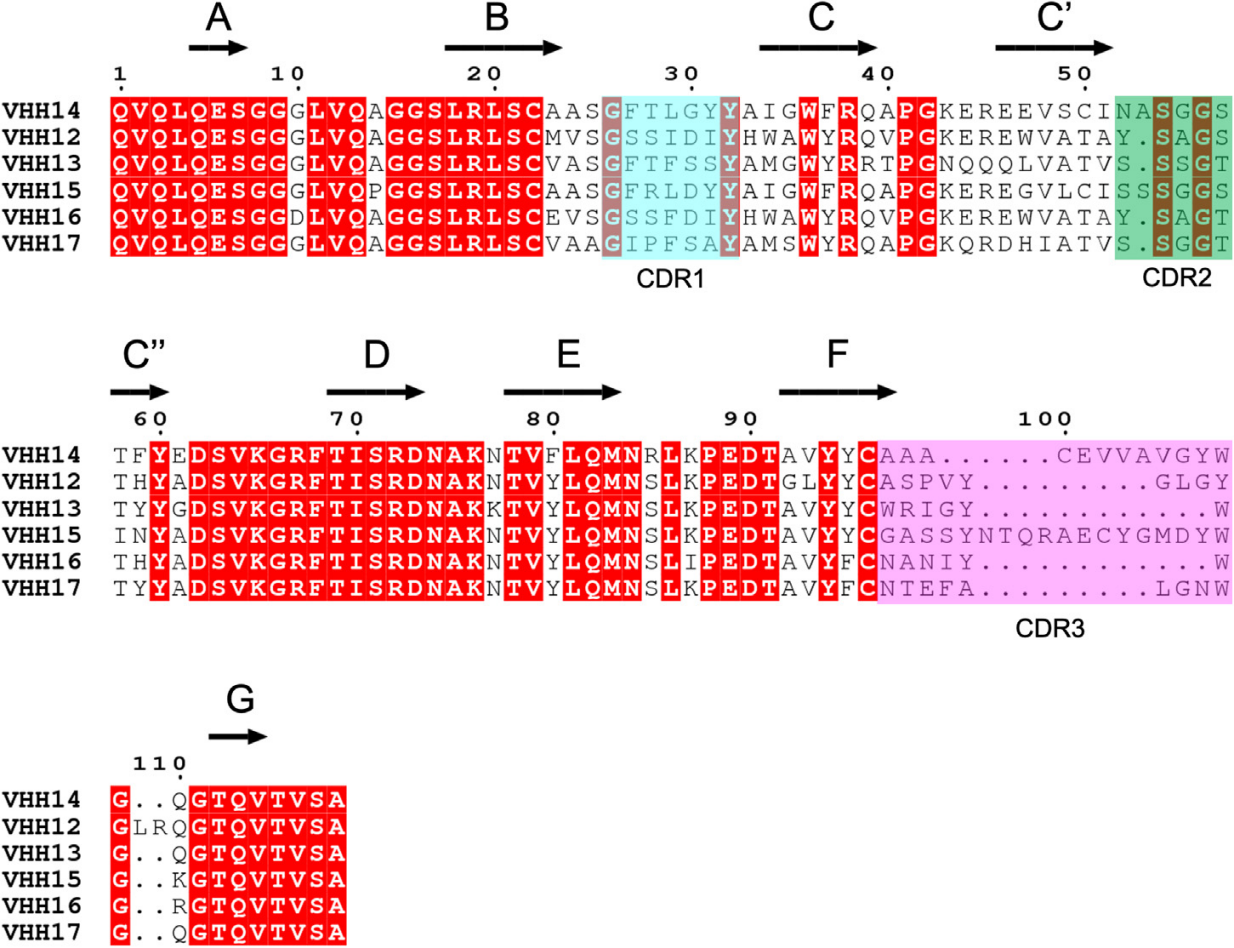
**A multiple sequence alignment of the most common VHH sequence seen for the six identified CDR3 families of anti-Plexin-B1 VHHs.** The positions of CDR1, CDR2, and CDR3 are indicated, and the secondary structure determined for VHH14 is shown above the alignment for reference, as are the β-strand identifiers. The CDRs are highlighted in *cyan* for CDR1, *green* for CDR2, and *magenta* for CDR3. Conserved residues are highlighted with *red boxes*. The figure was prepared using ESPript 3. 0 (45).

Ninety-six random clones from each of the second and third rounds of selection were sequenced. Sequence analysis revealed ten unique CDR3 sequences from six distinct families (Fig. 2). Sequences encoding the most frequent VHH clone within each CDR3 sequence were recloned into a mammalian expression vector upstream of the sequence encoding the Fc of human IgG1. A duplicate of one of the CDR3 sequences, with an unusual long CDR1, was also included. The resulting VHH-Fc fusion proteins were expressed in Expi293F cells with the correct size of the secreted VHH-Fc confirmed by SDS-PAGE (Fig. S1).

**Figure 3:**
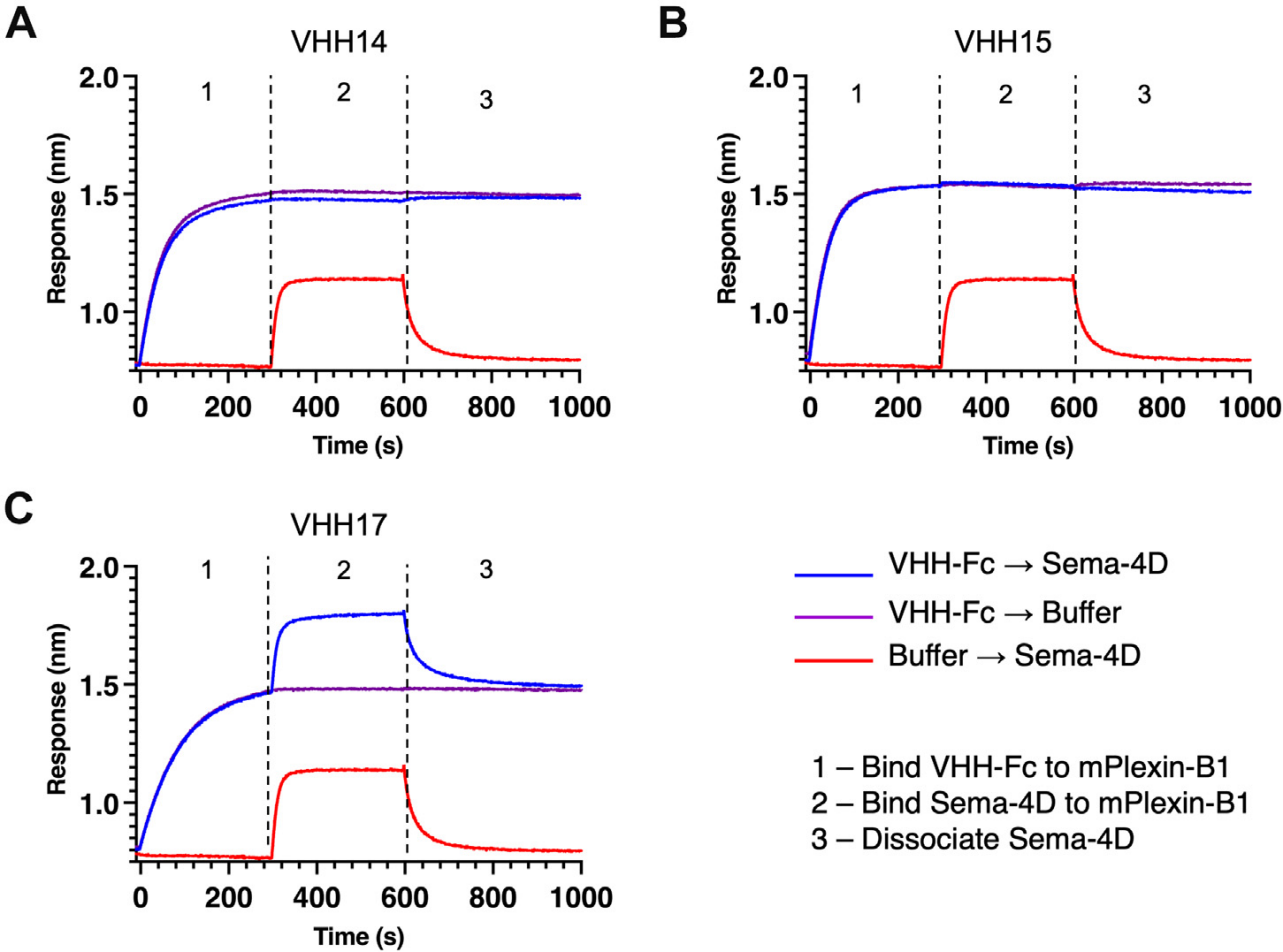
**VHH14 and VHH15 inhibit Sema4D binding to mouse Plexin-B1.** Typical sensorgrams are shown for VHH-Fc/Sema4D competition assays carried out using Octet RED384 BLI instrument. Biotinylated mouse Plexin-B1 was captured using streptavidin (SA) biosensors, and incubated with different combinations of VHHs, Sema4D, and buffer. The three phases of the assay are indicated for each trace: 1—bind VHH-Fc to immobilized mouse Plexin-B1 (20–535), 2—incubation with either Sema4D plus VHH-Fc; Sema4D only or VHH-Fc only, 3—dissociation of Sema4D in the presence or absence of VHH-Fc as per step 2. For each trace, colors indicate which proteins were present during stages 1 and 2, *blue*—VHH-Fc (1) followed by Sema4D plus VHH-Fc (2); *purple*—VHH-Fc (1) followed by VHH-Fc (2); and *red*—buffer (1) followed by Sema4D only (2). *A*–*C*, the panels show the traces for the following VHH-Fc fusions (*A*) VHH14-Fc, (*B*) VHH15-Fc, and (*C*) representative noninhibitory VHH (VHH17-Fc). The initial mouse Plexin-B1 load step has been omitted.

**Figure 4:**
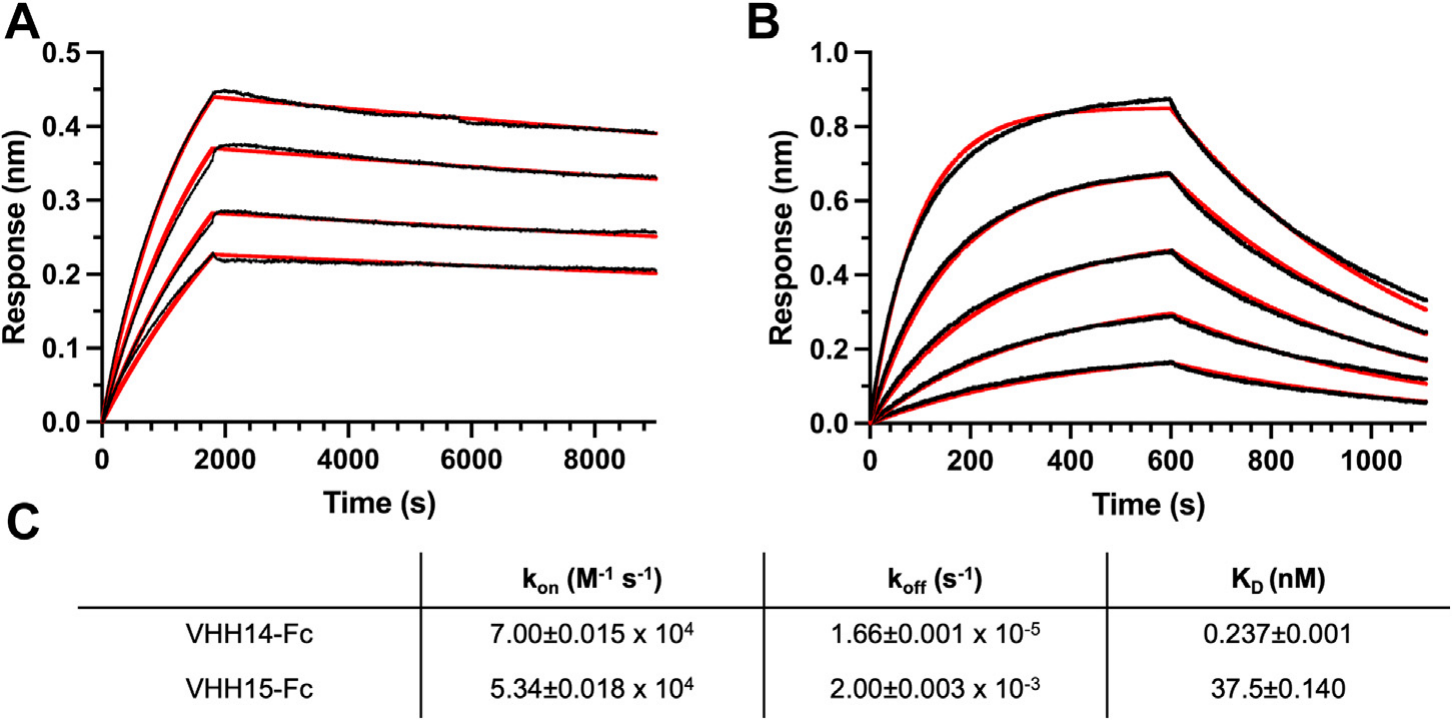
**Kinetics of mouse Plexin-B1 binding to VHH14-Fc and VHH15-Fc.** The kinetics of the inhibitory VHH-Fcs binding to mouse Plexin-B1 (20–535) were determined using an Octet RED384 BLI instrument. VHH-Fcs were captured using ProteinG biosensors and incubated with mouse Plexin-B1 at varying concentrations. Data collection times and mouse Plexin-B1 concentrations were optimized for each VHH-Fc using preliminary data. *A*, VHH14-Fc binding was assayed at mouse Plexin-B1 concentrations of 10 nM, 7.5 nM, 5 nM, and 3.75 nM. *B*, VHH15-Fc binding was assayed at 160 nM, 80 nM, 40 nM, 20 nM, and 10 nM. All traces are shown with relevant reference biosensors (without VHH-Fc bound) subtracted. *C*, kinetics parameters determined using global analysis, with a 1:1 binding model in ForteBio Data Analysis V 11.1 Parameters determined are quoted with standard error. Fits are shown in panels *A* and *B* as *red traces*, with reference subtracted sensorgrams in *black*.

Supernatants containing VHH-Fcs were tested for binding to mouse and human Plexin-B1 by BioLayer Interferometry. Ten out of 11 VHH-Fcs–bound mouse Plexin-B1 (Fig. S2*B*), whereas most of them did not recognize human Plexin-B1, with the exception of VHH16-2, which interacted weakly with human Plexin-B1 (Fig. S2*A*). VHH12 did not bind mouse Plexin-B1 in the BLI experiments. Two VHH-Fc efficiently blocked Sema4D binding to mouse Plexin-B1, identified as VHH14 and VHH15 (Fig. 3). VHH14-Fc and VHH15-Fc were subsequently purified. The binding kinetics of their interaction with mouse Plexin-B1 was characterized, and K_D_ values of 0.237 ± 0.001 nM and 37.5 ± 0.14 nM respectively were determined (Fig. 4). VHH14 exhibits much slower off rates than VHH15; however, both VHHs show off rates within the range reported for antibodies of similar K_D_ (28). It was also identified that VHH14 and VHH15 were capable of binding simultaneously to mouse Plexin-B1 (Fig. S3).

### COS-7 collapse assay

**Figure 5:**
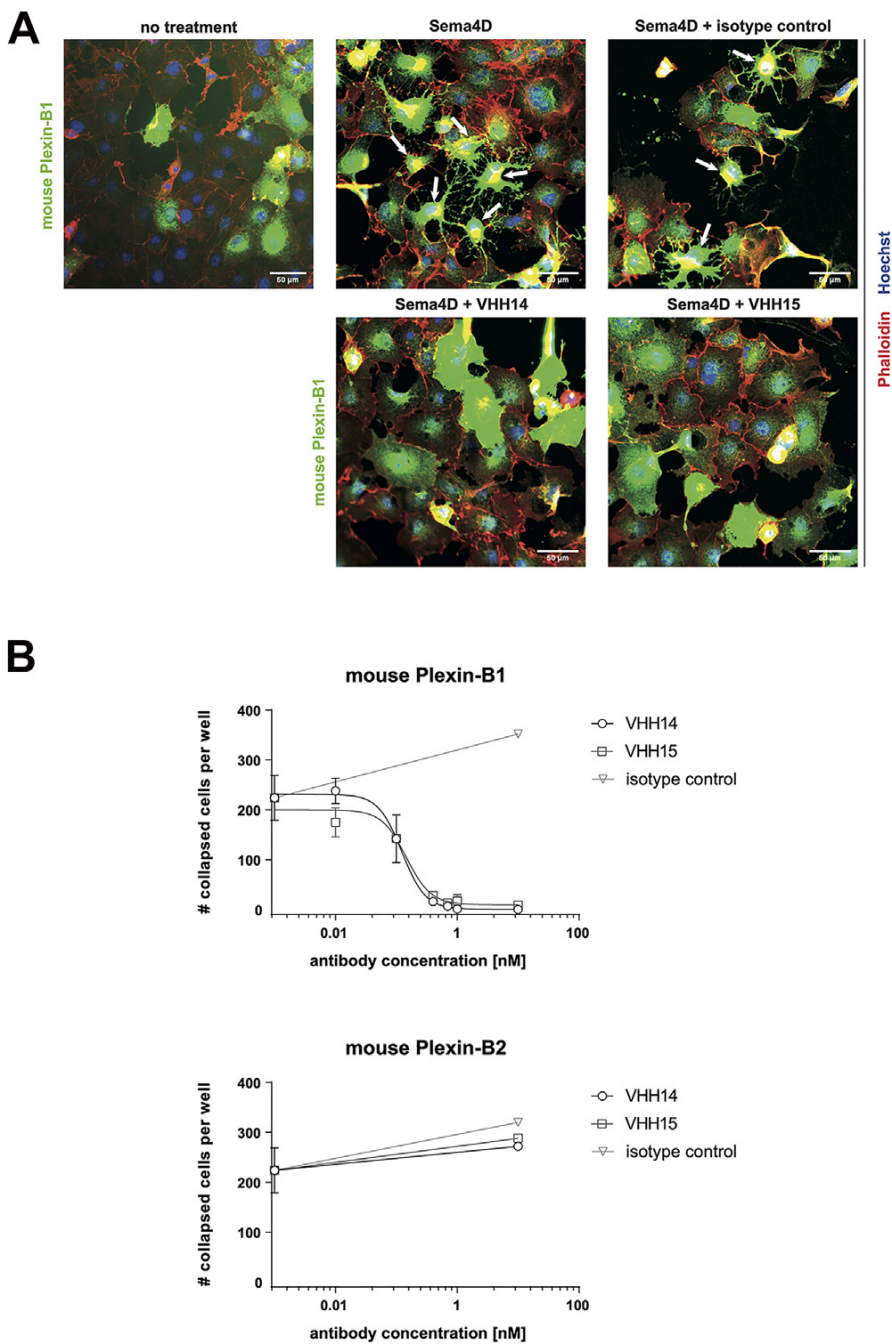
**VHH14-Fc and VHH15-Fc inhibit Sema4D-induced COS-7 cell collapse.***A*, COS-7 cells were transfected with recombinant FLAG-tagged mouse Plexin-B1 or mouse Plexin-B2. Forty-eight hours after cDNA transfection, cells were treated with VHH14-Fc, VHH15-Fc, or a nonbinding VHH-Fc (VHH12-Fc) as an isotype control, followed by treatment with or without Sema4D (50 nM for mouse Plexin-B1 expressing cells, and 150 nM for mouse Plexin-B2 expressing cells). Representative fluorescence images of immunostainings using anti-FLAG antibody (*green*) on mouse Plexin-B1 expressing cells are shown. Examples of collapsed cells are indicated with *white arrows,* and the scale bar represents 50 μm. *B*, COS-7 cells were transfected as described in (*A*), treated with VHH14-Fc, VHH-15Fc, or VHH-Fc isotype control at the indicated concentrations followed by Sema4D. Cellular collapse was determined as described in Experimental procedures. Shown are representative examples of two independent experiments (biological replicates). Graphs depict mean values ± SD.

The biological activity of the nanobodies identified was assessed using a COS-7 collapse assay (29), which revealed the ability of the nanobodies to block Sema4D-induced actin rearrangements in COS-7 cells transfected with murine plexin receptors. Labeling of the FLAG epitope of the transfected receptors was used to identify Plexin-B-expressing cells, and Sema4D dose–response experiments were used to confirm the activity of the Plexin-B-FLAG constructs and Sema4D. Both VHH14-Fc and VHH15-Fc were able to inhibit the Sema4D–induced collapse of COS-7 cells, with IC_50_s of 0.15 ± 0.04 nM and 0.09 ± 0.09 nM, respectively (Fig. 5, *A* and *B*).

The specificity of the VHHs for mouse Plexin-B1 was demonstrated in the same system. Neither VHH14-Fc nor VHH15-Fc was able to inhibit Sema4D-induced cell collapse for cells transfected with mouse Plexin-B2 (Fig. 5*B*). Human plexins were not tested, since the nanobodies were known to not bind human Plexin-B1 from BLI experiments (Fig. S2*A*). The lack of activity of the isotype control VHH-Fc indicated that the inhibition of plexin signaling was solely due to the VHH domain of VHH14-Fc and VHH15-Fc.

### Structure of the mPlexin-B1:VHH15 complex

To identify the mechanism of inhibition of Sema4D binding to mPlexin-B1 by VHH15, the structure of the complex of VHH15 with mPlexin-B1 was solved by X-ray crystallography. Initial crystals of the mPlexin-B1:VHH15 complex did not diffract to high resolution; however, supplementation of the crystallization buffer with 1% (w/v) dextran sulfate (M_r_ 5000) resulted in a change of crystal morphology, and these crystals diffracted to 2.95 Å (Table 1).

**Table 1:** X-ray data and refinement statistics

Crystal	VHH15: mPlexin-B1	VHH14:VHH15: mPlexin-B1	hPlexin-B1
Data collection			
Beamline	BESSY II 14.1	ESRF ID23-2	Diamond I04
Wavelength (Å)	0.918400	0.873127	0.97949
Space group	P 1 2_1_ 1	P 1 2_1_ 1	I 4_1_ 2 2
Cell dimensions			
a, b, c (Å)	93.5, 109.0, 94.0	95.3, 109.2, 95.5	266.7, 266.7, 108.7
α, β, γ (^o^)	90.0, 90.0, 90.0	90.0, 90.7, 90.0	90.0, 90.0, 90.0
Resolution (Å)	47.148–2.948	95.471–2.143	188.580–2.685
R_meas_	0.445 (2.161)	0.127 (1.089)	0.328 (1.737)
I/σI	5.9 (1.3)	7.4 (1.4)	7.4 (1.7)
CC_1/2_	0.953 (0.368)	0.984 (0.234)	0.994 (0.805)
Completeness (Spherical, %)	84.9 (27.0)	71.9 (16.2)	69.8 (16.8)
Completeness (Ellipsoidal, %)	93.4 (54.1)	93.3 (57.3)	94.6 (65.1)
Redundancy	6.4 (7.1)	3.4 (3.4)	13.5 (13.7)
No. of reflections	218,686 (13,470)	261,058 (13,915)	513,932 (43,483)
No. of unique reflections	33,937 (1890)	77,163 (4602)	38,131 (3178)
Wilson *B*-factors (Å)	36.92	32.33	34.54
Refinement			
R_factor_/R_free_ (%)	20.5/23.1	21.2/22.7	19.6/23.9
*B*-factors (Å)			
Protein	43.23	47.42	47.35
Solvent	31.71	42.81	44.27
R.M.S. Deviations			
Bond lengths (Å)	0.007	0.005	0.011
Bond angles (°)	1.11	0.80	1.30
Ramachandran plot			
Most favored (%)	92.4	95.6	94.1
Allowed (%)	6.67	3.89	5.80
Outliers (%)	0.93	0.50	0.10

Values in parentheses are for highest-resolution shell.

The crystals contained two copies of the complex of mPlexin-B1 with VHH15 in the asymmetric unit. The two copies were highly similar, with an overall Cα RMSD of 0.754 Å between the mPlexin-B1 monomers and 0.475 Å between the VHH15 monomers, respectively. The structure of mPlexin-B1 was also highly similar to the published structure of human Plexin-B1 bound to Sema4D (PDB:3OL2), with a Cα RMSD of 1.55/1.24 Å for the A and B chains, respectively, as expected given the high sequence identity of 87.6% between the human and murine domains 1 and 2. The majority of the nanobody is well defined in the complex, with the exception of the C-terminal 6xHis tag. CDR1, CDR2, and CDR3 are all well-defined, with an additional disulfide bond seen between Cys50 and Cys108, serving to anchor CDR3 to the framework on the C′ strand. CDR2 is composed exclusively of serine and glycine residues and does not interact with mPlexin-B1.

**Figure 6:**
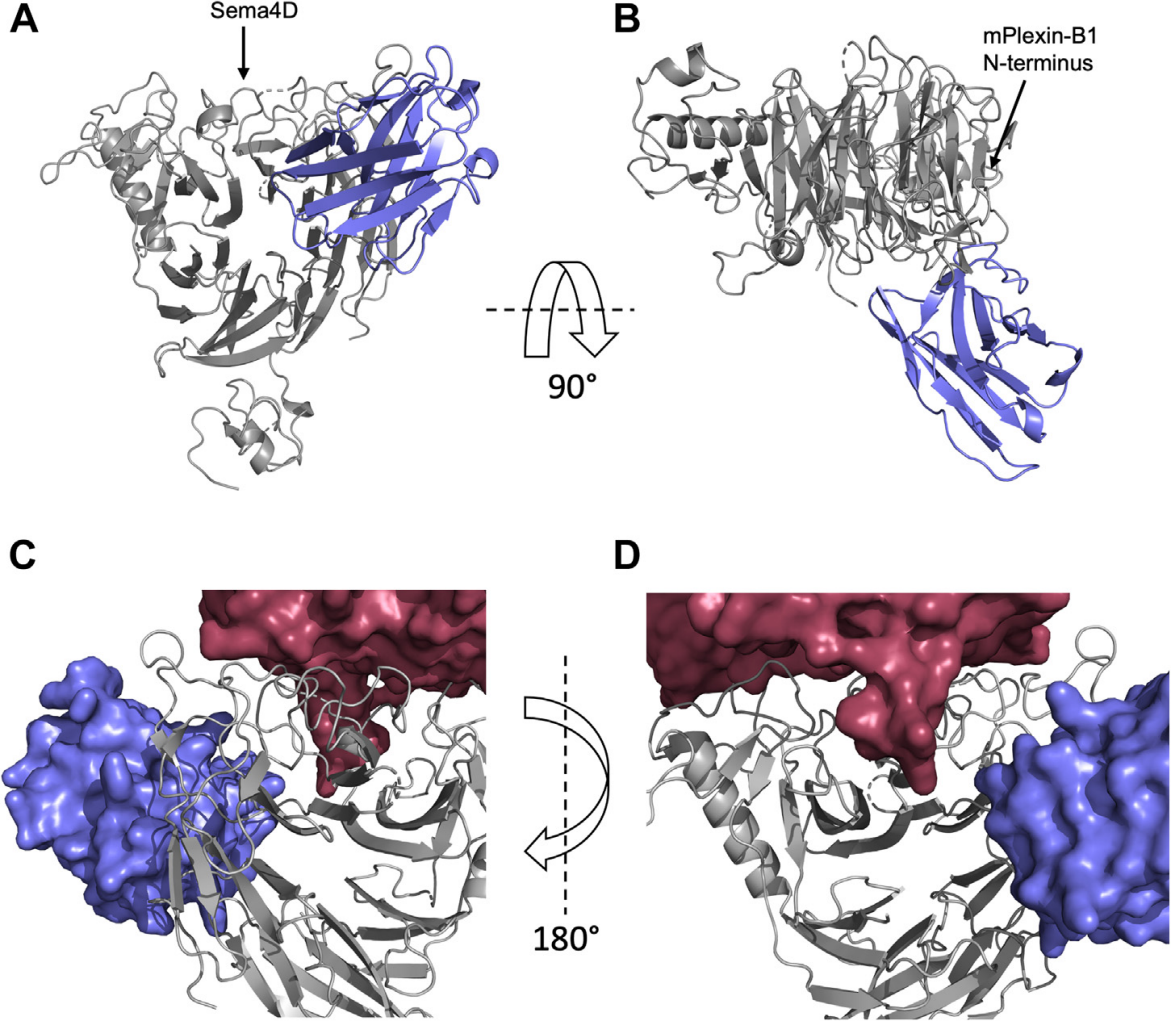
**Overall structure of the VHH15:mPlexin-B1 complex.** The complex of mouse Plexin-B1 (20–535) with VHH15 is shown from a front (*A*) and top (*B*) view relative to the sema domain. The topologies of the two proteins are shown as ribbon representations, with mPlexin-B1 in *gray* and VHH15 in *blue*. *A* and *B*, the locations of the Sema4D binding site (*A*) and the N terminus of mPlexin-B1 (*B*) are indicated with *arrows*. *C* and *D*, VHH15 bound to mPlexin-B1 does not overlap with Sema4D bound to hPlexin-B1. The published structure of Sema4D bound to human Plexin-B1 (pdb:3OL2) was aligned to the sema domain of mPlexin-B1 in complex with VHH15. Sema4D and VHH15 are shown as surface views in *red* and *blue*, respectively, with mPlexin-B1 shown in a *ribbon representation*. There is no visible overlap between Sema4D and VHH15, suggesting that the inhibition of Sema4D binding occurs through an allosteric mechanism.

**Figure 7:**
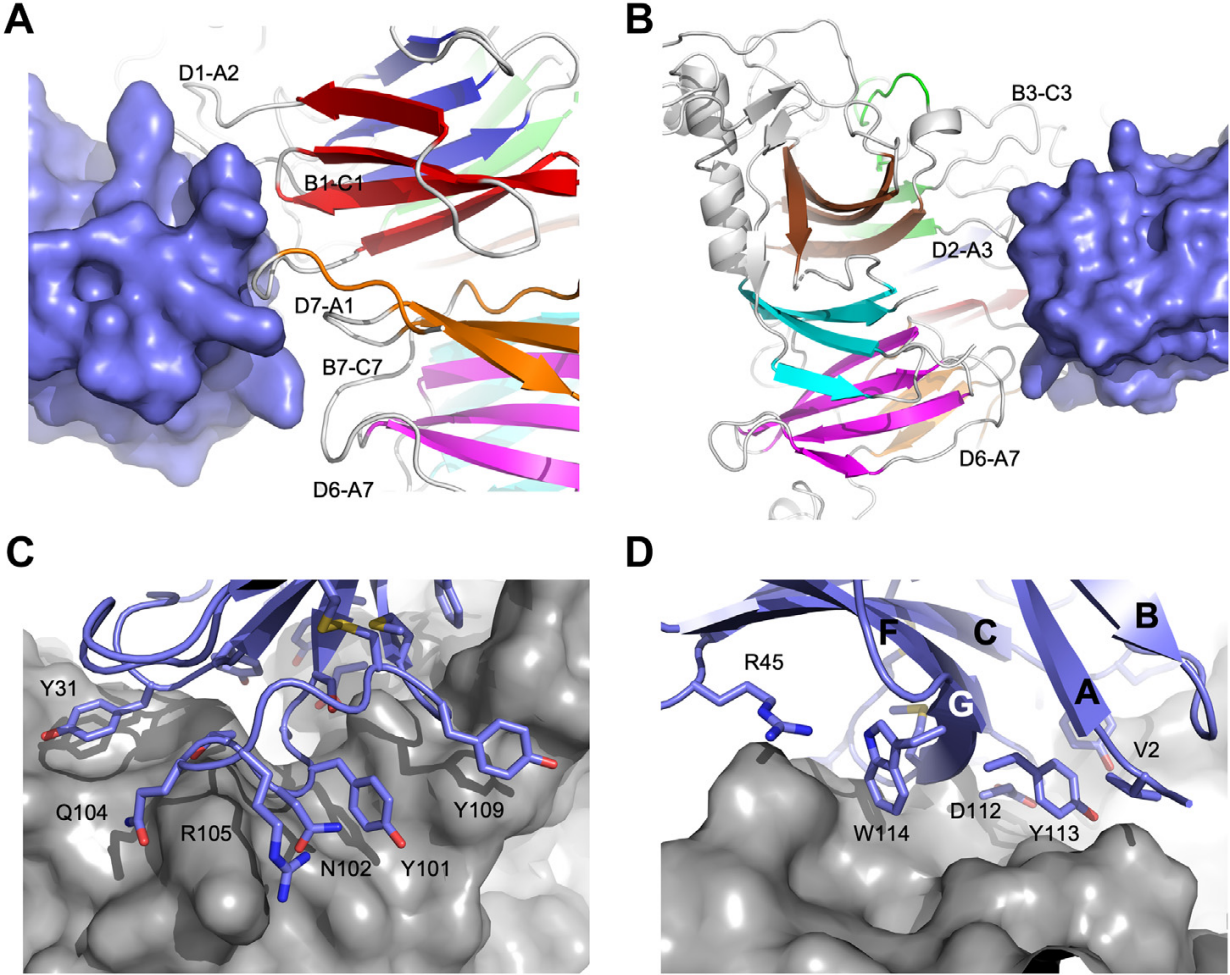
**Details of the interactions between VHH15 and mPlexin-B1.** The regions of VHH15 and mPlexin-B1 that interact were identified by a PISA analysis of the complex. *A* and *B*, the regions of VHH15 identified are shown as a *ribbon representation* (*A* and *B*) contacting a surface view of mPlexin-B1. Residues identified as being involved in contacts with Plexin-B1 have their side chains shown with both residues and β-strands labeled. *C* and *D*, the regions of mPlexin-B1 that are involved in the interface with VHH15 are also *highlighted* (*C* and *D*) with their loops labeled according to the β-strands of the sema domain connected. The β-strands of Plexin-B1 are colored as per Figure 1, with loops and insert regions of the sema domain in *gray*. The sema domain is shown as a *ribbon representation* with VHH15 shown as a *blue surface representation*.

The interface of mPlexin-B1 with VHH15 buries 892/904 Å^2^ for chains A and C, respectively, or 4.2% of the total surface area of mPlexin-B1 (20–535). VHH15 binds to one face of the sema domain of mPlexin-B1 (Fig. 6, *A* and *B*), on the opposite face to the N terminus, and does not overlap the Sema4D binding site which is located on the top surface of the semaphorin domain (18, Fig. 6*A*). When the structure of human Plexin-B1 bound to Sema4D is compared to the structure of VHH15 bound to mouse Plexin-B1, it can be seen that there is no overlap between Sema4D and VHH15 (Fig. 6, *C* and *D*) indicating that the VHH probably inhibits the binding of Sema4D in an allosteric manner. The nanobody interacts with blades 1, 2, 3, and 7 of the semaphorin domain (Fig. 1*A*), specifically the D7-A1 loop, B1-C1 loop, D1-A2 insertion, D2-A3 loop, B3-C3 loop, D6-A7 loop, and B7-C7 loop (Fig. 7, *A* and *B*).

The mPlexin-B1 interaction surface on VHH15 is mainly composed of residues from CDR1 and CDR3, together with residues from the N terminus, and the C′ as well as the G strand (Fig. 7, *C* and *D*). Specifically, the interface involves Val2 from the N terminus, Gly26, Tyr31, and Tyr32 from CDR1, Arg45 from the C′ strand, Ser99, to Met111 from CDR3 and Asp112, Tyr113 and Trp114 from the G strand. The interaction surface buries 871/891 Å^2^ or 13.1%/13.5% of the surface area of the two copies of VHH15 present, chains B and D, respectively.

Analysis of the sequence differences in the sema domains of human and murine Plexin-B1 (Fig. 1*B*) identified several amino acids differences, which were in close proximity to the binding interface of VHH15 with mPlexin-B1 and could contribute to the selectivity of VHH15 for murine Plexin-B1 over human. Visual inspection of the overlayed structures identified His34, which forms a hydrogen bond between the side chain and the backbone carbonyl of Tyr31 in CDR1 as being of interest. This residue is a tyrosine in human Plexin-B1, and inspection of the crystal structure indicated that the side chain of the tyrosine in human Plexin-B1 would clash with CDR1 of VHH15 in the complex (Fig. S4*A*). To test the importance of this sequence difference, a H34Y P240Q mutant of mPlexin-B1 was assayed for binding to VHH15 *via* biolayer interferometry and was found to show minimal detectable binding to mPlexin-B1 (Fig. S5*D*). This indicates that this single amino acid change is largely responsible for the selectivity of VHH15 for murine over human Plexin-B1.

### Structure of the mPlexin-B1:VHH14:VHH15 complex

Attempts to obtain a well-diffracting crystal of VHH14 bound to mPlexin-B1 (20–535) were not successful, so a ternary complex of both VHH14 and VHH15 with mPlexin-B1 was pursued. Epitope binning experiments indicated that both VHH14 and VHH15 were capable of simultaneously binding to mPlexin-B1 (Fig. S3). Size-exclusion chromatography and SDS-PAGE confirmed that VHH15 was able to bind with high affinity to the VHH14:mPlexin-B1 complex, and crystals were obtained for this ternary complex that diffracted to 2.15 Å (Table 1). Analysis of the diffraction data identified that two copies of the ternary complex of mPlexin-B1 (20–535) with VHH14 and VHH15 was present in the asymmetric unit.

**Figure 8:**
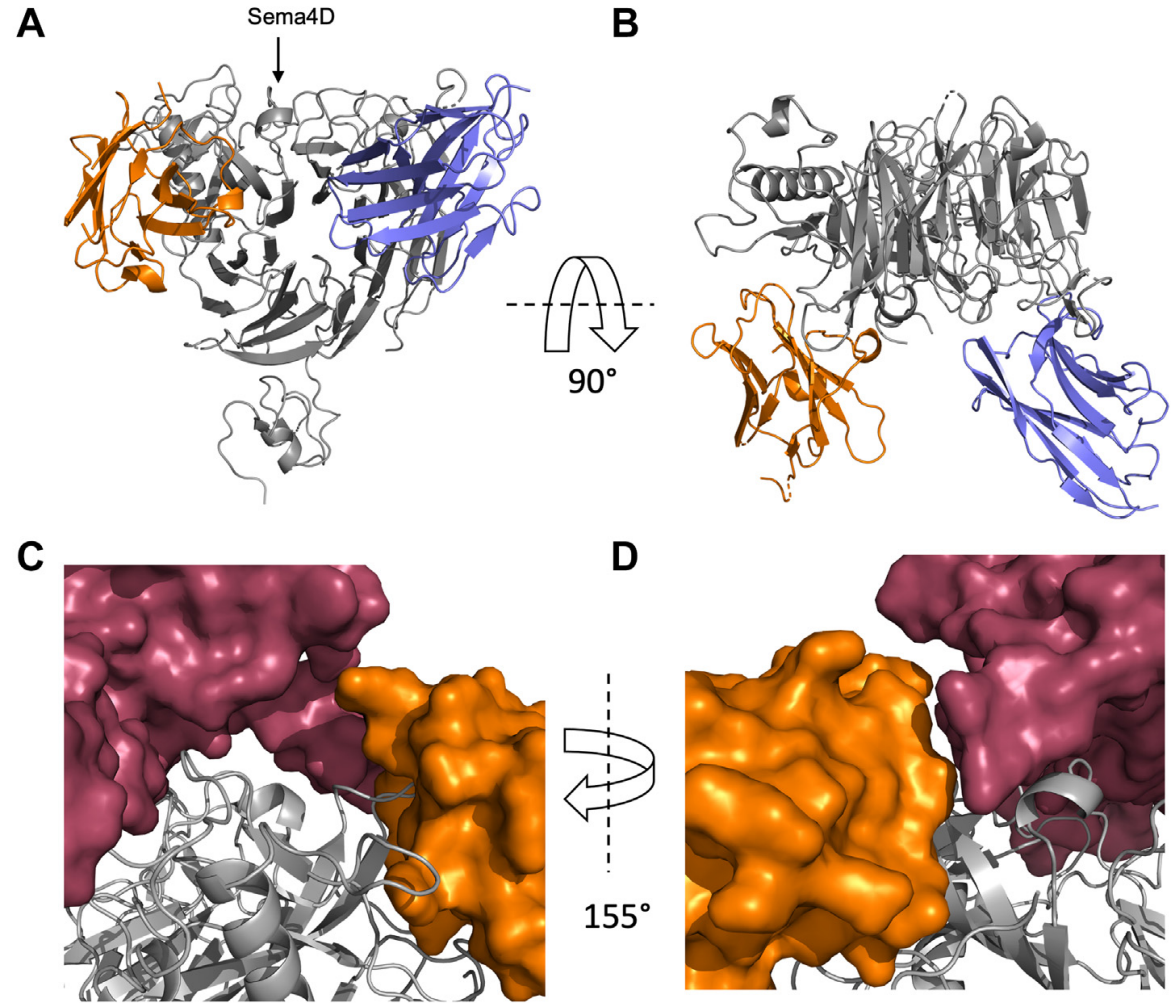
**Overall structure of the VHH15:VHH14:mPlexin-B1 ternary complex.***A* and *B*, the complex of mouse Plexin-B1 (20–535) with VHH14 and VHH15 is shown from a front (*A*) and top (*B*) view relative to the sema domain. The topologies of the two proteins are shown as *ribbon representations*, with mPlexin-B1 in *gray*, VHH14 in *orange,* and VHH15 in *blue*. The location of the Sema4D binding site is indicated with an *arrow*. *C* and *D*, VHH14 bound to mPlexin-B1 does not significantly overlap with Sema4D bound to hPlexin-B1. The published structure of Sema4D bound to human Plexin-B1 (pdb:3OL2) was aligned to the sema domain of mPlexin-B1 in complex with VHH14 and VHH15. Sema4D and VHH14 are shown as surface views, in *red* and *orange* respectively, with mPlexin-B1 shown in a *ribbon representation*. Although regions of VHH14 and Sema4D are close (*D*), there is no overlap of the proteins, suggesting that inhibition of Sema4D binding may occur though an allosteric mechanism.

The binding interface of mPlexin-B1 with VHH15 was essentially unchanged from the 2.95 Å structure of the mPlexin-B1:VHH15 complex. As in the binary complex, VHH15 was well defined in the electron density, with the entire protein chain visible, apart from the C-terminal 6xHis tag. VHH14 was found to bind to mPlexin-B1 *via* the sema domain, on the same face as VHH15 (Fig. 8, *A* and *B*), but on the opposite side of that face to VHH15. Comparison of Sema4D bound to human Plexin-B1 with VHH14 bound to mouse Plexin-B1 showed that there was no overlap between Sema4D and VHH14 when the sema domains of Plexin-B1 were aligned (Fig. 8, *C* and *D*). Whilst CDR3 is found in close proximity to residues 348 to 352 in human Sema4D (Fig. 8*D*), and may, partially interfere with the binding of Sema4D, overall, the suggestions that the mechanism of Sema4D-binding inhibition by VHH14 is likely to be allosteric.

The structure of mPlexin-B1 in the ternary complex is highly similar between the two copies of the protein in the asymmetric unit, with a Cα RMSD of 0.723 Å. The two are also highly similar to the structure of mPlexin-B1 found in the VHH15 complex, with a Cα RMSD of 0.763 Å between chain C and its most similar counterpart in the VHH15 structure (chain A), and 0.689 Å between chain F and its most similar chain in the VHH15 structure (chain B). The overall conformation of the sema domain of plexin has not changed between the single and double complex, and this is confirmed by a visual examination of the structural alignment of the different mPlexin-B1 chains. Some subtle changes are observed, particularly in the loops contacting VHH14, but the binding interface with Sema4D is essentially unchanged between the two complexes.

**Figure 9:**
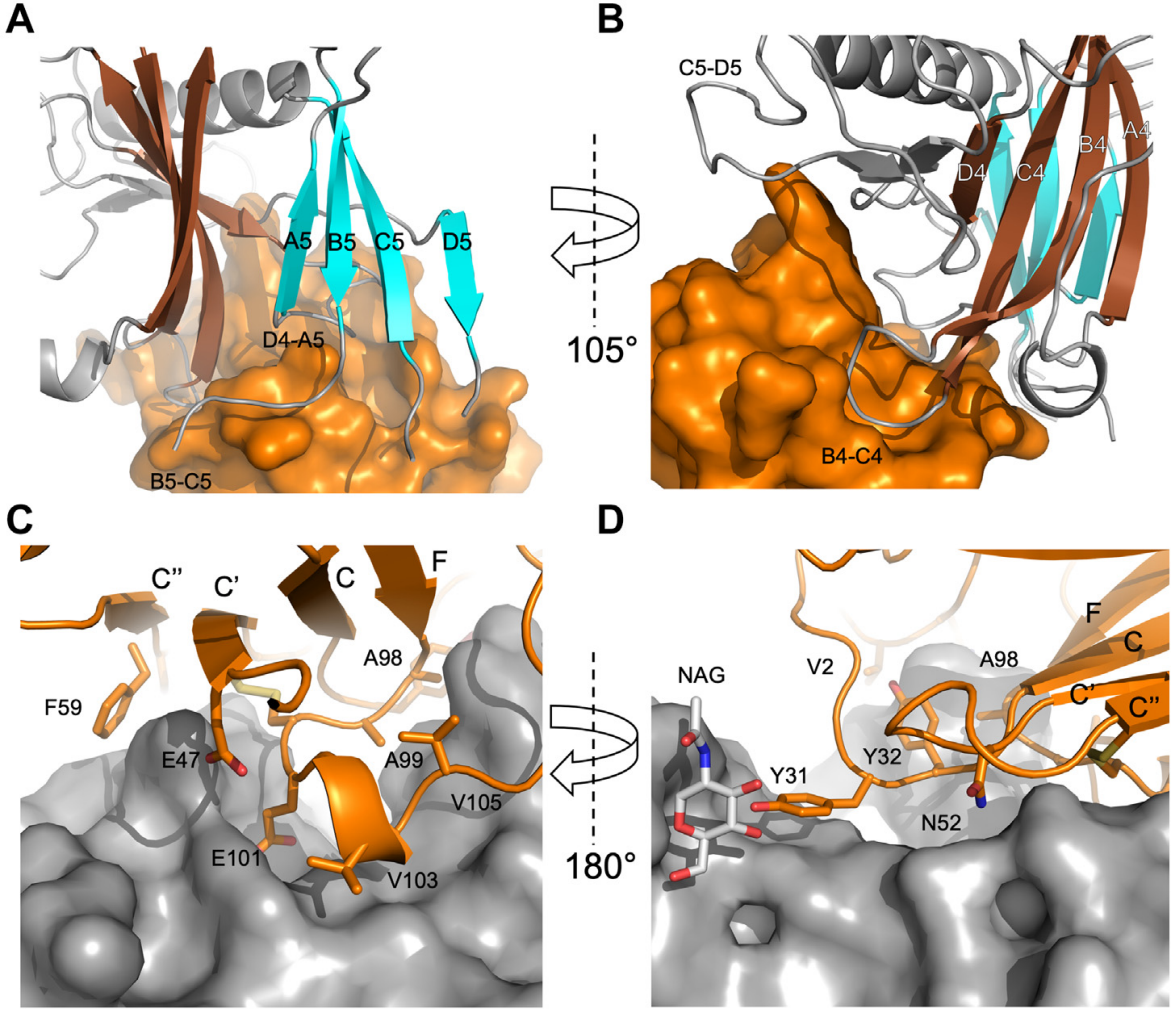
**Details of the interactions between VHH14 and mPlexin-B1.** The regions of VHH14 and mPlexin-B1 that interact were identified by a PISA analysis of the VHH14:VHH15:mPlexin-B1 complex. *A* and *B*, the loop regions of mPlexin-B1 that are involved in the interface are labeled (*A* and *B*) according to the β-strands of the sema domain connected. The β-strands of Plexin-B1 are colored with blade 4 in *brown* and blade 5 in *cyan*, with loops and insert regions of the sema domain in *gray*. The sema domain shown as a *ribbon representation,* and VHH15 is shown colored *orange* and as a surface representation. *C* and *D*, the regions of VHH15 identified as involved in the complex are shown on a *ribbon representation* in *orange* (*C* and *D*) against a surface view of mPlexin-B1 in *gray*. Residues identified as being involved in contacts have their side chains shown, and the residues and β-strand are identified. The N-acetylglucosamine attached to Asn349 is also shown separately to the rest of mPlexin-B1.

The interaction of VHH14 with mPlexin-B1 buries 922/910 Å^2^ on mPlexin-B1 chains C and F, respectively, or 4.1% of the surface of mPlexin-B1 D1-2. The total buried surface of the interface is very similar between VHH14 and VHH15 and is consistent with the average areas of nanobody:target interfaces (30). The interaction of VHH14 is with blades 4 and 5 of the sema domain, as well as with the long insert between blades 5 and 6. Specifically, the nanobody contacts the B4-C4 loop, the D4-A5 loop, the B5-C5 loop, and the C5-D5 insertion (Fig. 9, *A* and *B*). The nanobody also interacts with the N-linked glycan found on N349 through Y31 (Fig. 9*D*), with the N-acetylglucosamine moiety, and the side chain of Y31 both well defined in the electron density (Fig. S6). More extensive interactions are likely to occur with high-mannose or native glycans due to the proximity of the visible sugar and the vector by which the glycan would be extended. However, for structural analysis, the N-linked glycans present on mPlexin-B1 were trimmed to a single N-acetylglucosamine by EndoH to aid crystallization.

The interaction surface on VHH14 involves contributions from all three CDR loops, as well as contributions from the N terminus. CDR3 binds into the grove between blades 4 and 5 of the sema domain, with residues A98, A99, E101, V103, and V105, contributing to this section of the interface. CDR1 interacts with the C5-D5 insertion, primarily though Y31 interacting with the N349 glycan (Fig. 9, *C* and *D*). The majority of VHH14 is well defined for both chains present, with a single region of poor density, Gly9-Gly16 in chain A. This region is found at the base of the nanobody and is not close to the paratope region. The interaction buries 913/923 Å^2^ on chains A and D, respectively, consisting of 13.9%/14.2% of the total surface area of VHH14.

Relatively few of the sequence differences that occur between the sema domain of murine and human Plexin-B1 (Fig. 1*B*) are found near the interface of VHH14 and mPlexin-B1; however, one nonconserved amino acid was identified by visual inspection of the structure as a potentially critical residue to the selectivity of VHH14 for murine Plexin-B1. Proline-240 is found in the center of the B4-C4 loop of mPlexin-B1, which binds in a cleft between the N terminus, CDR1 and CDR3. This residue is a glutamine in human Plexin-B1, which was postulated to interfere with the binding of VHH14 (Fig. S4*B*). This was tested through assessing the binding of mPlexin-B1 (20–535) H34Y P240Q to VHH14 *via* BLI (Fig. S5, *A* and *B*). This revealed in a K_D_ for the interaction of 255 nM compared to 3.13 nM WT mPlexin-B1 under the same conditions (Fig. S5*E*), suggesting that this residue is important for the selectivity of VHH14 for mPlexin-B1 over hPlexin-B1. The relatively lower effect of this mutation, compared to the H34Y mutation, may indicate that the N349 glycan makes a significant contribution to the affinity of VHH14 to mPlexin-B1 and the selectivity for mPlexin-B1 over hPlexin-B1. This glycan is absent in human Plexin-B1, as the asparagine to which the glycan is attached in mPlexin-B1 is an aspartate in hPlexin-B1 (Fig. 1*B*). The side chain of N349 in mouse Plexin-B1 is positioned over 6 Å from VHH14, and the side chain of D349 in human Plexin-B1 is positioned even further from the VHH14 binding site, indicating that the lack of the glycan in human is the key difference as opposed to the asparagine to aspartate change as significant structural re-arrangement would need to occur to allow VHH14 to contact this residue.

### Structure of human Plexin-B1

To further understand the mechanism of the VHHs inhibition of Sema4D binding to Plexin-B1, the structure of human Plexin-B1 (20–535) was solved by X-ray crystallography to a resolution of 2.69 Å (Table 1), with the asymmetric unit containing two copies of hPlexin-B1. Crystals of murine Plexin-B1 were also obtained in the same conditions as those of human Plexin-B1, but these did not diffract to a suitable resolution for structure determination.

The overall structure of hPlexin-B1 was similar to the published structure of Sema4D bound hPlexin-B1 (PDB:3OL2, Fig. 1*A*), with a Cα RMSD of 1.87/1.80 Å for chains A and B, respectively. When only the sema domains are considered, the Cα RMSD drops to 1.72/1.67 Å. These Cα RMSDs are greater than those observed for mPlexin-B1 from the VHH15 complex compared to the Sema4D bound structure of human Plexin-B1. Several cadmium ions were found in the crystal structure, some of which support the crystal packing observed.

### Structural changes in human Plexin-B1 on Sema4D binding

Previously reported structures of the Plexin-A2:Sema6A and Plexin-B1:Sema4D complexes, when compared to the apo Plexin-A2, Sema6A and Sema4D structures (18, 20) suggested that the sema domain of Plexin-B1 did not undergo any conformational changes on Sema4D binding, since the Plexin-A2 and Sema6A sema domains did not undergo such a change nor did the Sema4D sema domain.

Initial Cα RMSD comparisons of the two chains found in the apo human Plexin-B1 structure revealed that the PSI domain (480–535) undergoes significant loop movements within the crystal form, as do several loops in the sema domain (Fig. S7). This inherent flexibility of the PSI domain can also be seen in the published human Plexin-B1:PB1m6 complex structure (21), whereby the flexibility in this domain meant that it was only modeled in one out of the six copies of Plexin-B1 present. In addition, several of the loops in this domain are poorly defined in the electron density for both published structures, such as Jannsen *et al.* (2010) and the structures reported here, suggesting that the loops in this domain are flexible. For this reason, changes in the PSI1 domain between published and herein reported structures were not considered in the analysis.

**Figure 10:**
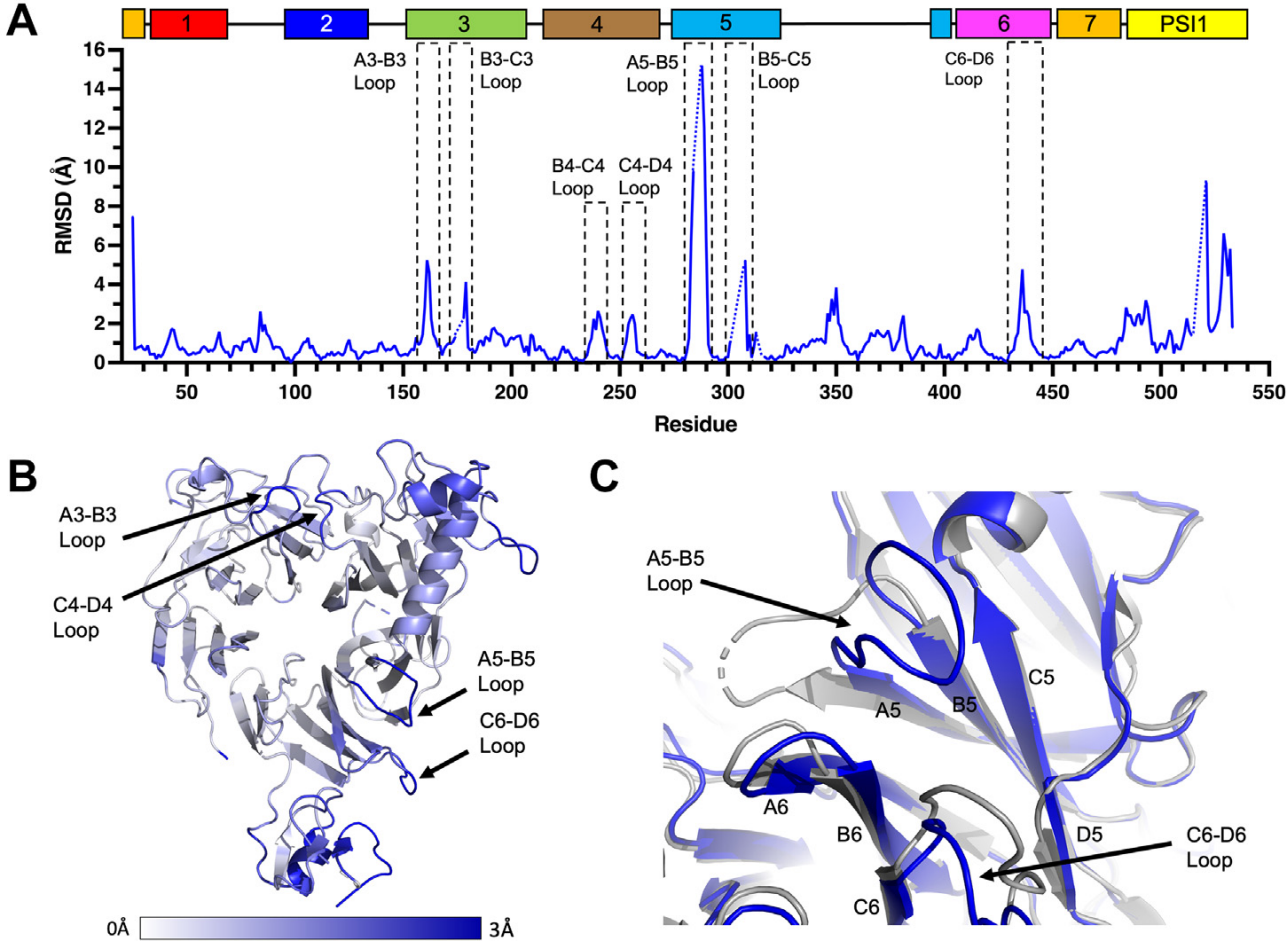
**Conformational changes occur in human Plexin-B1 on Sema4D binding.***A*, pairwise Cα RMSDs were calculated between the structures of Sema4D-bound human Plexin-B1 and apo human Plexin-B1 chain A. This allowed the identification of regions of human Plexin-B1 (20–535) that underwent significant changes on Sema4D binding. Regions for which either structure did not contain a residue are indicated with a *dotted line*. The location of the seven blades of the sema domain and PSI1 domain are indicated above the plot, and the position of key loops which undergo substantial shifts between apo and Sema4D-bound states are also highlighted with *dashed boxes*. *B*, the Cα RMSD between PDB:3OL2 chain B and human Plexin-B1 chain A is highlighted on a *ribbon representation* of human Plexin-B1 which reveals that backbone movements extend around the entire sema domain of human Plexin-B1. Cα RMSDs are shown as a gradient from 0 to 3 Å. *C*, *ribbon representation* of a region of human Plexin-B1 for the apo protein (*blue*) and Sema4D-bound (*gray*) structures illustrating the substantial movement of the A5-B5 loop in human Plexin-B1 and the differences in the position of the C6-D6 loop induced by Sema4D binding. The β-strand IDs surrounding this region are indicated on the strands.

These conformational differences seen between the two copies of apo hPlexin-B1 present were used as reference points for the conformational changes observed between the apo and the Sema4D bound form. Contrary to what has been reported for Plexin-A2 (18), our analysis revealed significant loop movements in Plexin-B1 induced by Sema4D binding, which extend beyond the binding interface of Sema4D (Fig. 10, *A* and *B*). These form concerted movements that are transmitted through the sema domain, including subtle twisting motions of the beta strands and associated blades of the sema domain. The largest movements seen are up to 15.2 Å in the position of the A5-B5 loop (Fig. 10*A*). In the apo structure, this loop is folded over and interacts with the cleft between blades 5 and 6 of the sema domain (Fig. 10*C*). This region in the structure is the site to which the allosteric inhibitory peptide PB1m6 peptide binds to which was previously thought to be a preformed cleft in the sema domain (21). Although not all of this loop is modeled in the Sema4D-bound structure, most of the loop is found within the deposited electron density (Fig. S8*A*), and the whole loop is visible within the structure of apo hPlexin-B1 reported here (Fig. S8*B*). This loop varies significantly in length as well as sequence between the different plexin subclasses. The length of the loop between the A5 and B5 strands varies from 19 residues in murine Plexin-A2 to 11 residues in human Plexin-B1 and six residues in Plexin-C1. The intermediate loop length in Plexin-B1 provides the flexibility and length to allow these conformational changes between the apo and bound states, without forming the more stable, additional secondary structure seen in Plexin-A2.

Regions of increased RMSD also identify that conformational changes are seen in several loops on both faces of the sema domain (Fig. 10*A*), such as the A3-B3 loop, the B3-C3 loop, B4-C4 loop, C4-D4 loop, B5-C5 loop, and the C6-D6 loop. The C6-D6 loop is adjacent to the cleft formed by the motion of the A5-B5 loop in the Sema4D bound structure and is further retracted from this region in the apo structure, presumably to allow space for the insertion of the A5-B5 loop in the apo structure reported here (Fig. 10*C*). These additional changes are smaller in magnitude than those seen in the A5-B5 loop and do not occur in regions of the Plexin-B1 sema domain known to be involved in ligand or coreceptor binding.

Subsequent to the determination of the structure of apo human Plexin-B1 (20–535), a model of full-length human Plexin-B1 was determined by AlphaFold (31, 32). The conformation of the sema domain of Plexin-B1 in the model is more similar to the apo structure reported here, than to the deposited, Sema4D-bound structure (PDB:3OL2). In the C6-D6 and A5-B5 loop regions, the AlphaFold model shows a similar closed cleft to the apo structure, albeit with the C6-D6 loop inserting further into the cleft, and the A5-B5 loop in a more open conformation than the experimentally determined structure (Fig. S9).

### Allosteric nanobodies induce a Sema4D-bound like conformation of Plexin-B1

**Figure 11:**
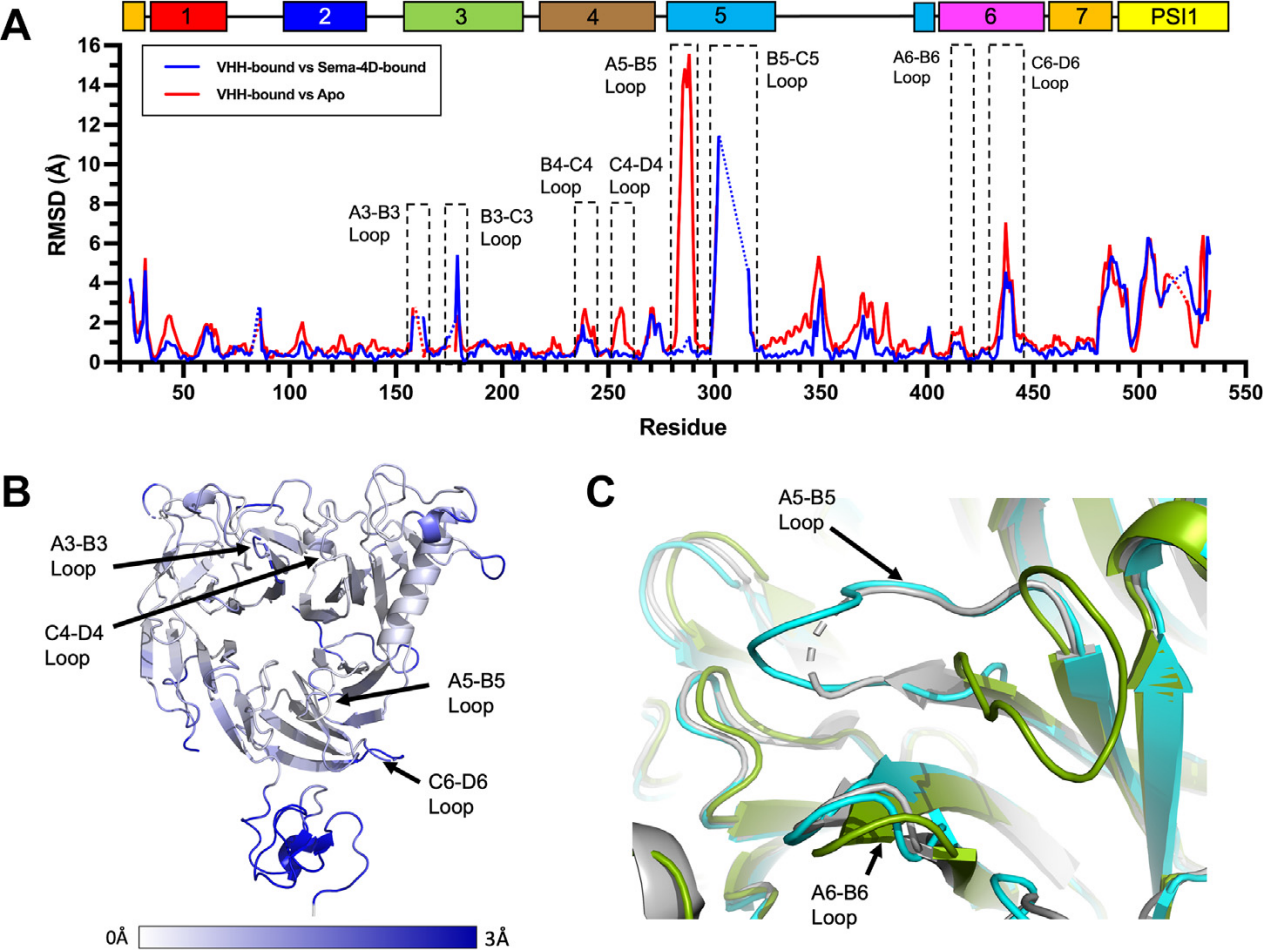
**Anti-Plexin-B1 VHHs induce a Sema4D bound-like conformation in mouse Plexin-B1.***A*, pairwise Cα RMSDs were calculated to identify regions of mouse Plexin-B1 (20–535) that underwent significant changes on VHH binding and compare to the Sema4D bound conformation of human Plexin-B1. The better defined mouse Plexin-B1 chain (chain C) was compared to the human Plexin-B1 component of PDB:3OL2 (*blue*) and the same chain of mouse Plexin-B1 with the best defined human Plexin-B1 chain (chain A, *red*). Residues which were not present in either compared structure are indicated with a *dotted line*. The location of several key loops are indicated by *dashed boxes*. The A5-B5 loop is seen to have a lower RMSD between VHH14:VHH15 and Sema4D bound Plexin-B1 than between VHH14:VHH15 bound and apo human Plexin-B1. The location of the blades of the sema domain and the PSI1 domain are indicated above the plot. Numbering is by full-length human Plexin-B1. *B*, the Cα RMSD between PDB:3OL2 chain B and VHH14:VHH15:mPlexin-B1 chain C is shown on a *ribbon representation* of apo human Plexin-B1 revealing substantially fewer conformational changes on the sema domain of human Plexin-B1 compared to the conformational changes seen between apo and Sema4D-bound human Plexin-B1 (Fig. 11*B*), although substantial changes still occur in the PSI1 domain. Cα RMSDs are shown as gradient from 0 to 3 Å. The location of several loops which showed significant shifts between apo and Sema4D-bound hPlexin-B1 is indicated. *C*, The A5-B5 loop region in the VHH-bound form of mouse Plexin-B1 adopts a Sema4D bound-like conformation, as illustrated by the ribbon representations for the Sema4D bound form of human Plexin-B1 (*gray*, PDB:3OL2), the apo form of human Plexin-B1 (*green*), and the VHH14- and VHH15-bound form of mouse Plexin-B1 (*cyan*).

In order to understand the allosteric mechanism of VHH14 and VHH15 inhibition of Sema4D binding, the structure of the sema domain of murine Plexin-B1 bound to both nanobodies was compared to the Sema4D bound and apo conformations of human Plexin-B1. Similar to the changes observed between the apo structures, a substantial variation was observed in the PSI domain (Fig. 11*A*). As with the apo human Plexin-B1 structure, these changes were considered to be due to the inherent flexibility of the PSI domain and its interface with the sema domain.

Subtle changes in the positioning of the loops involved in the binding interface of Plexin-B1 with Sema4D were observed with the nanobody-bound mPlexin-B1. The degree to which these changes are responsible for the inhibition of Sema4D binding to Plexin-B1 is not clear, since these changes are small and would not cause apparent steric clashes between Sema4D and nanobody-bound Plexin-B1 (Fig. S10). Interestingly, the A5-B5 loop, which was observed to undergo a significant conformational change between the apo and Sema4D-bound Plexin-B1 (Fig. 10*C*), was seen to adopt a conformation similar to that observed in the Sema4D-bound structure (Fig. 11*C*). A movement of up to 7.89 Å (Fig. 11*A*) was also observed in the B5-C5 loop. This loop interacts directly with VHH14, *via* CDR3, but the tip of the loop remains highly flexible and not visible in the electron density, as with the apo structures. In the VHH15 only complex, this loop is also flexible and is not visible in the electron density. The movements in this loop in the VHH14 complex may be involved in the inhibitory mechanism of that nanobody but do not appear to be required for the action of VHH15.

The two inhibitory nanobodies appear to induce a mouse Plexin-B1 a conformation similar to the Sema4D-bound structure (18) and to that seen with an inhibitory allosteric macrocycle (21). Stabilization of this conformation, by either a macrocycle or nanobody, results in the affinity of Plexin-B1 for Sema4D being significantly reduced, causing an inhibition of signaling.

## Discussion

Previous structural studies of plexins and semaphorins have concluded that their ligand binding sema domains are relatively rigid structures (18), with the signaling following semaphorin binding mediated through changes in the oligomeric state of plexin on the cell surface (17).

The work presented here questions this paradigm by showing that the sema domain of Plexin-B1 undergoes significant structural rearrangement upon semaphorin binding (Fig. 10). This suggests that different classes of plexin receptors may adopt differing mechanisms of regulation and/or signal transmission on ligand binding. Previous studies had identified the allosteric regulation potential of the Plexin-B1 sema domain (21), but the impact of these observations had been limited by the absence of an apo Plexin-B1 structure, which made the mechanism of action of the allosteric peptide PB1m6 difficult to ascertain.

The structures of the nanobody complexes presented here reveal that the sema domain of Plexin-B1 has multiple allosteric sites. Indeed, had further inhibitory nanobodies been identified, additional allosteric sites may well have been discovered, since the structural changes that occur on the binding of allosteric inhibitors, as well as ligand binding, extend around the entire sema domain (Figs. 10*B* and 11*B*). It is particularly interesting that all of the allosteric inhibitors of Sema4D binding discovered to date bind to a Sema4D-bound like conformation of the sema domain of Plexin-B1. The bound conformation seen in the crystal structure is likely to be, therefore, a low-affinity conformation, perhaps allowing for the release of bound Sema4D from the complex.

Overall, the results and analysis presented here indicate that the sema domain of Plexin-B1 is primed for allosteric regulation *via* conformational changes and that these conformational changes are key for transmission of signals from semaphorin binding to activate the intracellular domain of Plexin-B1. This opens multiple new avenues to explore the biological activity of Plexin-Bs and suggests multiple options for the pharmaceutical modulation of Plexin-B1 activity.

## Experimental procedures

### Generation of recombinant Plexin-B1 constructs

A DNA sequence encoding humanPlexin-B1 (1–535) or corresponding residues of mouse Plexin-B1 (1–535) was cloned into a pcDNA5 vector with the addition of a C-terminal 6xHis-tag. The plasmids were transfected into Expi293F cells (Invitrogen) according to manufacturer’s instructions. Supernatant containing secreted Plexin-B1 (residues 20–535) was collected after 7 days and purified using two-step purification protocol. Briefly, secreted protein was captured on an Excel HisTrap (GE HealthCare) column. After washing the column with 10 column volumes of 20 mM imidazole, the protein was eluted with 250 mM imidazole. This was followed with gel filtration chromatography using a Superdex 200 16/600 column equilibrated with PBS. Protein containing fractions were analyzed on SDS-PAGE, pooled, quantified by A_280_, aliquoted, and flash-frozen for storage at −80 °C. Purified human and murine Plexin-B1 (20–535) were analyzed by mass spectrometry and SEC-MALS to confirm identity and quality. An Avi-tagged version of mouse Plexin-B1 (20–535) was generated by insertion of the Avi-tag (GLNDIFEAQKIEWHE) between the C terminus of the Plexin-B1 fragment and the 6xHis-tag using the QuikChange Lightning Site-Directed Mutagenesis Kit (Agilent Technologies). The resulting mouse Plexin-B1 (20–535)-Avi-6xHis construct was expressed and purified using the same method as described for nonmodified version. The *in vitro* biotinylation of the Avi-tagged protein was carried out using the BirA enzyme (BirA500 standard reaction kit, Avidity) according to the manufacturer’s protocol. Free biotin was removed by a desalting method using Zeba Spin 7K desalting columns (ThermoFisher). The biotinylation was verified by Western blotting analysis using Streptavidin-HRP conjugate (N100, ThermoFisher). Recombinant Sema4D (1–677) with C-terminal 6xHis-tag in a pcDNA5 vector was expressed, purified, and analyzed using the method described for Plexin-B1 constructs above.

### Huarizo immunization with mouse Plexin-B1

An adult huarizo was subjected to four subcutaneous injections, each containing 300 μg of purified mouse Plexin-B1(20–535)-6xHis protein in emulsion of Freund’s adjuvant (CFA/IFA). The injections were administered at day 1, 28, 42, and 56 over a total period of 60 days (Preclinics). Serum from blood samples collected on day 0, 52, and 60 were titered by binding ELISA to determine the antigen-specific immune response.

### VHH library generation

Peripheral blood mononuclear cells isolated from 100 ml of blood collected on day 60 from the immunized animal in RNAlater (Invitrogen) were used for total RNA extraction using a RNeasy Midi kit (Qiagen). Two microgram of the total RNA was converted to cDNA using the Superscript III First-strand synthesis kit (Invitrogen) with a CH2 domain gene-specific primer. Amplification of variable regions of heavy-chain immunoglobulins (VHHs) from the cDNA was performed by 2-stage PCR, as described by Ghassabeh *et al.* (33) followed by ligation into the pHEN1H6 vector. The purified ligation mix was electroporated into TG1 cells (Lucigen) and cells plated onto 2xYTAG (2xYT agar + 2% (w/v) glucose) plates and grown overnight at 32 °C. In parallel, a serial dilution of the transformation mixture was plated on 2xYTAG plates to determine the total library size. The cells were scraped from the plates in liquid 2xYTAG, mixed with sterile glycerol to a final concentration of 20%, aliquoted, and stored at −80 °C. Colony PCR and Sanger sequencing were carried out on 96 random colonies to quality control the final library.

### Phage production and library panning

Bacteriophage expressing anti-Plexin-B1 VHH were produced by infecting the bacterial library with M13K07 helper phage and were precipitated from culture media with 20% (w/v) PEG 8000, 2.5 M NaCl, and resuspended in PBS. Three rounds of selection using murine Plexin-B1 (20–535) were performed. The outputs from each selection round were used to infect exponential growing TG1 cells which were plated on agar plates. Colonies from the second and third rounds were sequenced and high-frequency CDRs identified using AptaAnalyser (34).

### Cloning, expression, and purification of VHH-Fc and VHH-6xHis fusions

Plasmid DNA encoding the most frequent clone within each VHH CDR3 family was purified using the QIAprep Spin Miniprep Kit (Qiagen) following manufacturer’s instructions. VHH encoding sequences were subcloned by ligation-independent cloning into the pHCMVi-Script HuIgG1 Fc vector from pHEN1H6 plasmids encoding the VHHs. Incorporation of the correct insert into the pHCMVi vector was confirmed by DNA sequencing.

Expi293F cells were transfected with 1 μg of a pHCMVi-Script plasmid encoding VHH-Fc in a 24-well plate and 1 ml final volume using the Expi293 expression system kit (Gibco) and following the manufacturer’s instructions. Five days posttransfection the cells were centrifuged, and supernatants were collected. The production of a secreted VHH-Fc was verified by SDS-PAGE under reducing and nonreducing conditions. The amount of expressed VHH-Fc in each supernatant was quantified by using Protein G biosensors on an Octet RED384 instrument (ForteBio), calibrated to previously purified isotype control VHH-Fc.

VHH14-Fc and VHH15-Fc were subsequently expressed in 200 ml cultures of Expi293F cells as described above apart from using 200 μg plasmid DNA for transfection. The supernatants were collected 7 days posttransfection by centrifuging the culture and filtering the supernatant through a 0.22 μm filter unit. A two-step purification was performed for VHH14-Fc and VHH15-Fc. The supernatant was initially loaded onto a HiTrap MabSelect column (Cytiva) and eluted with 0.1 M sodium citrate pH 3.0. The eluted fractions were neutralized with 1 M Tris pH 9.2 prior to loading onto a HiLoad Superdex 200 26/600 pg column (GE Healthcare) equilibrated in PBS.

pHCMVi-Script plasmids encoding VHH14 and VHH15 with C-terminal 6xHis-tag were generated using In-Fusion cloning (Clontech) by PCR amplifying DNA fragments from the pHCMVi-Script-VHH-Fc plasmids and introducing a 6xHis-tag. Correct incorporation of the insert and 6xHis-tag was confirmed by DNA sequencing.

VHH14-6xHis and VHH15-6xHis were also expressed in Expi293F cells following transfection with pHCMVi-Script plasmid encoding VHH14-6xHis or VHH15-6xHis. VHH14-6xHis and VHH15-6xHis were purified from Expi293 supernatants by immobilized metal affinity and size-exclusion chromatography. The fractions containing purified protein were pooled and assessed by SDS-PAGE, with the identity of the purified protein confirmed by mass spectrometry and homogeneity of the purified protein confirmed by SEC-MALS.

### Analysis of mouse Plexin-B1 binding to VHH-Fc using biolayer interferometry

The binding experiments were carried out at 25 °C using an Octet RED384 (ForteBio). 1× Kinetics buffer (ForteBio) was used for dilutions and as the assay buffer. To characterize the interaction between mouse Plexin-B1 and purified VHH-Fcs, VHH14-Fc or VHH15-Fc were first immobilized on Protein G biosensors by incubating the sensors in 0.67 μg/ml VHH-Fc for 200s. The sensors were then incubated for 120s in the assay buffer to establish the baseline. VHH14-Fc loaded sensors were incubated in 10, 7.5, 5, and 3.75 nM mouse Plexin-B1 for 1800s followed by 7200s dissociation; VHH15-Fc loaded sensors were incubated in 1:2 serial dilutions of mouse Plexin-B1 starting from 160 nM for 600s followed by 510s dissociation. A control was run in parallel with a reference sensor with no VHH-Fc immobilized. For comparisons of mouse Plexin-B1 (20–535) wildtype and H34Y P240Q binding to VHH14-Fc and VHH15-Fc, VHH-Fc-loaded biosensors were incubated in 400 nM, 200 nM, 100 nM, 50 nM 25 nM, 12.5 nM and 6.25 nM mPlexin-B1 for 300s, followed by dissociation in assay buffer for 600s. Data were analyzed using the Kinetics module of the ForteBio Data Analysis software 11.1 and GraphPad Prism 9.4.1. Reference sensor data were subtracted from the ligand sensor data. Global analysis of multiple mouse Plexin-B1 concentrations and a 1:1 model was used to fit the data and obtain k_on_ and k_off_ values.

### Analysis of VHH-Fc inhibition of Sema4D binding to mouse Plexin-B1 using biolayer interferometry

VHH-Fc inhibition of Sema4D binding to Plexin-B1 was assayed using an Octet RED384 (ForteBio). 1× Kinetics buffer (ForteBio) was used for dilutions and as the assay buffer. Murine Plexin-B1 was immobilized on streptavidin biosensors by incubating the sensors in 50 nM biotinylated murine Plexin-B1 (25–535)-Avitag-6xHis for 300s, followed by 180s incubation of sensors in the assay buffer. The sensors were then transferred to 100 nM VHH-Fc for 300s, followed by either 10 μg/ml human Sema4D or assay buffer for 300s, and finally the assay buffer for 600s. In addition, a sensor without any VHH-Fc immobilized was included for detection of maximum Sema4D binding to murine Plexin-B1.

### COS-7 collapse assay

Full-length mouse Plexin-B1-FLAG and Plexin-B2-FLAG pcDNA3.1 plasmids were purchased from GenScript. COS-7 cells were purchased from ATCC and cultured in high-glucose Dulbecco’s modified Eagle’s medium supplemented with GlutaMAX, pyruvates (Gibco) and 10% fetal bovine serum (Gibco). Cells were cultured at 37 °C in 10% CO_2_ and passaged at 80 to 90%. COS-7 cells were then seeded in 96-well plates (Greiner) at 6500 cells per well in a total volume of 100 μl per well and transiently transfected 24 h later with plexin pcDNA3.1 plasmids, using the X-treme Gene 9 transfection reagent according to the manufacture instructions (Sigma).

After transfection for 48 h, anti-Plexin-B1 VHH14-Fc, VHH15 -Fc nanobodies, or the isotype control nanobody-Fc at a concentration of 0.01 to 10 nM (for Plexin-B1) or 150 nM (for Plexin-B2) were added, incubated for 1 h, and followed by an additional 1 h treatment with 50 nM Sema4D (for Plexin-B1) or 150 nM Sema4D (for Plexin-B2). Cells mock-treated with media instead of VHH-Fcs were incubated with 0.08 to 50 nM (Plexin-B1) or 10 to 150 nM (Plexin-B2) Sema4D for a Sema4D dose response to confirm correct Plexin-B activity following transfection.

Cells were fixed with 4% formaldehyde, permeabilized, and incubated with an anti-FLAG antibody (Sigma, F1804), followed by staining with an anti-mouse-Alexa Fluor 488 secondary antibody (Life Technology, A11001), Hoechst (Invitrogen, H3570), and Phalloidin Texas Red (Life Technology, T7471). After staining, the plates were imaged using a high-content screening system (IN Cell Analyser 2000, GE Healthcare). Images were analyzed and the number of collapsed cells per well was quantified manually. Nonlinear regression in GraphPad Prism was used to calculate IC_50_ values using a five-parameter equation.

### Preparation of VHH:Plexin-B1 complexes for crystallography

All single complexes of VHH-bound Plexin-B1 for crystallization were prepared by mixing a 20% molar excess of VHH with murine Plexin-B1, and 5000u of EndoH (NEB) in PBS, pH 7.4. This was dialyzed overnight at 4 °C against 25 mM Tris-HCl, pH 7.5, and 50 mM NaCl. The complex was then purified by size-exclusion chromatography using a Superdex 200 16/600 pg column (GE Healthcare), equilibrated in 25 mM Tris-HCl, pH 7.5, and 50 mM NaCl. Plexin-B1 apo samples were prepared using the same method, except that a Superdex 75 16/600 pg column (GE Healthcare) was used.

The ternary complex of VHH14 and VHH15 with murine Plexin-B1 was prepared by adding a 20% molar excess of VHH15 to purified VHH14:mPlexin-B1 complex, without the addition of further EndoH, followed by purification by size-exclusion chromatography, using a Superdex 200 16/600 pg column equilibrated in 25 mM Tris-HCl, pH7.5 and 50 mM NaCl. All complexes were concentrated to 10 mg/ml using Vivaspin centrifugal concentrators (Sartorius), flash-frozen using liquid N_2_, and stored at −80 °C.

### Protein crystallography of hPlexin-B1 and mPlexin-B1 complexes

Suitable protein concentrations for crystallization were identified using a Pre-Crystallization Test (PCT) (Hampton Research). Initial crystallization trials were performed using sitting-drop vapor diffusion and 96-well deep well block screens (Molecular Dimensions, Qiagen) at 18 °C. Crystal hits identified from screens were optimized in 15-well hanging drop plates (Qiagen).

For the VHH15:mPlexin-B1 complex, additive screening was performed using the Hampton Research Additive Screen (Hampton Research) at 18 °C using the original crystal hit condition. Batch-matched dextran-sulphate M_r_ 5000 (Hampton Research) was used to grow optimal crystals by hanging-drop vapor diffusion in 8% (w/v) PEG 8000, 0.1 M sodium citrate, pH 5.0, 1% (w/v) dextran-sulphate (M_r_ 5000) at a protein concentration of 5 mg/ml and 18 °C. A cryoprotectant of 22% ethylene glycol in mother liquor was used. X-ray diffraction data were collected at the BESSY II synchrotron (Berlin) using beamline 14.1 (35).

For the VHH14:VHH15:mPlexin-B1 complex, additive screening was also performed using the Hampton Research Additive Screen (Hampton Research) at 18 °C using the original crystal hit condition. Batch-matched dextran-sulphate M_r_ 5000 (Hampton Research) was used to grow optimal crystals by hanging-drop vapor diffusion in 15% (w/v) PEG 6000, 0.1 M sodium citrate, pH 5.5, 3% dextran sulphate (M_r_ 5000) at a protein concentration of 5 mg/ml and 18 °C. A cryoprotectant of 22% ethylene glycol in mother liquor was used. X-ray diffraction data were collected at the ESRF synchrotron (Grenoble) using beamline ID23-2.

Optimal crystals of human Plexin-B1 were grown in 1.0 M sodium acetate, 0.2 M cadmium sulphate, 0.1 M Hepes, pH 7.5, at a protein concentration of 5 mg/ml and 18 °C. The crystals were cryoprotected by increasing the sodium acetate concentration to 2.5 M, and diffraction data were collected at the Diamond Light Source (Oxford, UK) using beamline I04.

X-ray diffraction images were indexed and integrated with XDS (36) or DIALS (37) and scaled and merged using the STARANISO web server (38). All structures were determined by molecular replacement, using the program PHASER (39), with the following models used in the search.

For the VHH15:mPlexin-B1 complex, a model of the murine Plexin-B1 sema-PSI domain was prepared from the deposited human Plexin-B1: Sema4D complex structure (PDB:3OL2) using SWISS-MODEL (40). A homology model of VHH15 was generated using SWISS-MODEL, with the deposited nanobody structure 5LHN as the template. For the VHH14:VHH15:mPlexin-B1 complex, the already solved structure of the VHH15:mPlexin-B1 complex and a homology model of VHH14 prepared by SWISS-MODEL, using the closest sequence match deposited nanobody structure (PDB:5TOK) as a template, were used as the search models. For the hPlexin-B1 apo structure, the hPlexin-B1 component of the deposited structure of hPlexin-B1 bound to Sema4D (PDB:3OL2) was used as the search model.

Model building and structural refinement was carried out with Coot (41), REFMAC 5.8.0258 (42) and PHENIX-refine (43) using restrained refinement and isotropic B-factors. X-ray data collection and refinement statistics are given in Table 1. Structural biology software used in these studies was installed and configured by SBGrid (44).

All structural alignments were performed using Pymol (The PyMOL Molecular Graphics System, Version 2.3.2 Schrödinger, LLC), on Cα, with no cycles of outlier rejection performed. For sema domain only alignments, the final residue of the sema domain used was the conserved P478.

## Data availability

The VHH15:mPlexin-B1, VHH14:VHH15:mPlexin-B1 and hPlexin-B1 crystal structures reported here are available in the RSCB PDB under accession codes 8BB7, 8BF4 and 8B3K, respectively.

BioLayer Interferometry sensorgrams can be provided upon request.

## Supporting information

This article contains supporting information.

## Conflict of interest

The authors declare no conflicts of interest with the contents of this article.
