# Supplementary material for: Nanobody inhibitors of Plexin-B1 identify allostery in plexin–semaphorin interactions and signaling
Source: J Biol Chem. 2023 Apr 23;299(6):104740. doi: 10.1016/j.jbc.2023.104740 (PMC10206189; doi:10.1016/j.jbc.2023.104740)
Supplement: Supporting Figures S1–S10 [file mmc1.docx]

**Supplemental Figures.**

**Figure S1**

**
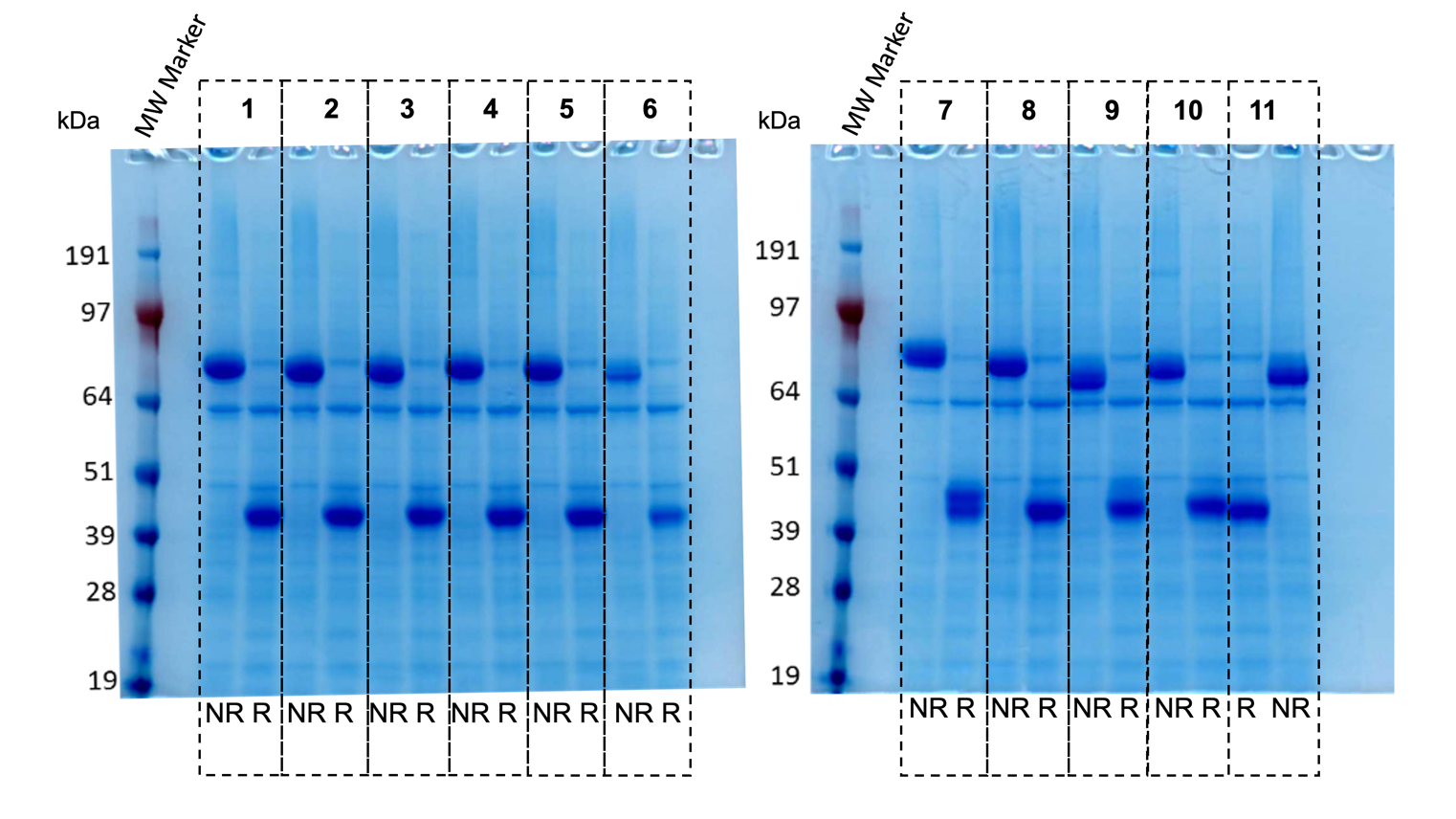
**

**Figure S1 SDS-PAGE analysis of the expression of VHH-Fc constructs**

Coomassie stained SDS-PAGE analysis of supernatants containing VHH-Fcs secreted from Expi293 cell cultures. Reduced (R) and non-reduced (NR) protein samples are indicated below the lanes. The protein molecular weight marker used was SeeBlue Plus2 (Invitrogen). The lanes for each VHH are indicated by a surrounding box, with the different VHHs distributed as follows: 1 – VHH16-1-Fc; 2 – VHH16-2-Fc; 3 – VHH16-3-Fc; 4 – VHH17-Fc; 5 – VHH12-Fc; 6 – VHH13-Fc; 7 – VHH16-4-Fc; 8 – VHH16-5-Fc; 9 – VHH14-Fc; 10 – VHH15-Fc and 11 – VHH16-Fc

**Figure S2**

**
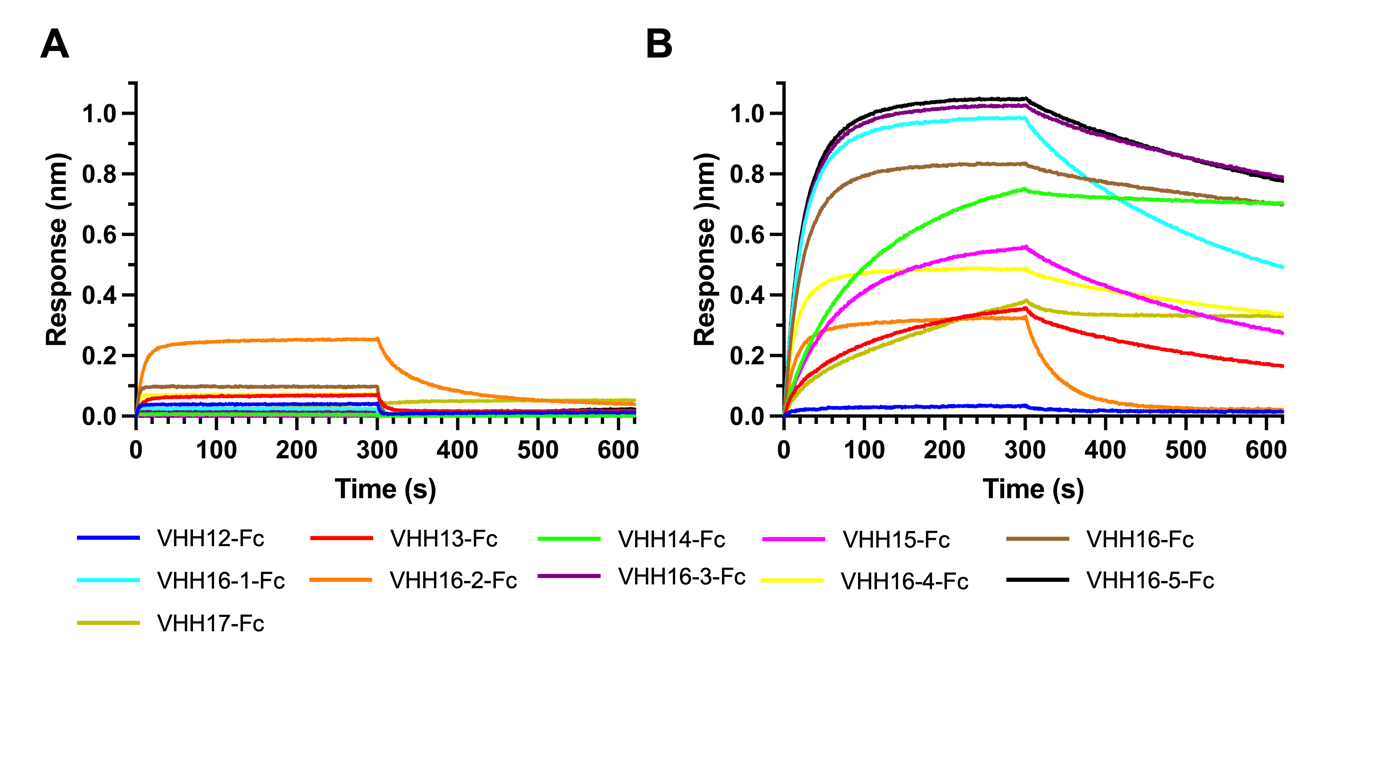
**

**Figure S2 The VHH-Fcs bind strongly to mouse Plexin-B1 but not to human Plexin-B1.** The ability of VHH-Fcs to bind Plexin-B1 was determined using an Octet RED384 BLI instrument. VHH-Fcs were captured with ProteinG biosensors and incubated with either (A) 10 μg/mL human Plexin-B1 (20-535) or (B) 10 μg/mL mouse Plexin-B1 (20-535). VHH-12 showed no detectable binding to either human or mouse Plexin-B1 and VHH16-2, which has an unusual long CDR1, shows binding to both human and mouse Plexin-B1, with a higher level of binding to mouse plexin. All sensorgrams are reference subtracted.

**Figure S3**

**
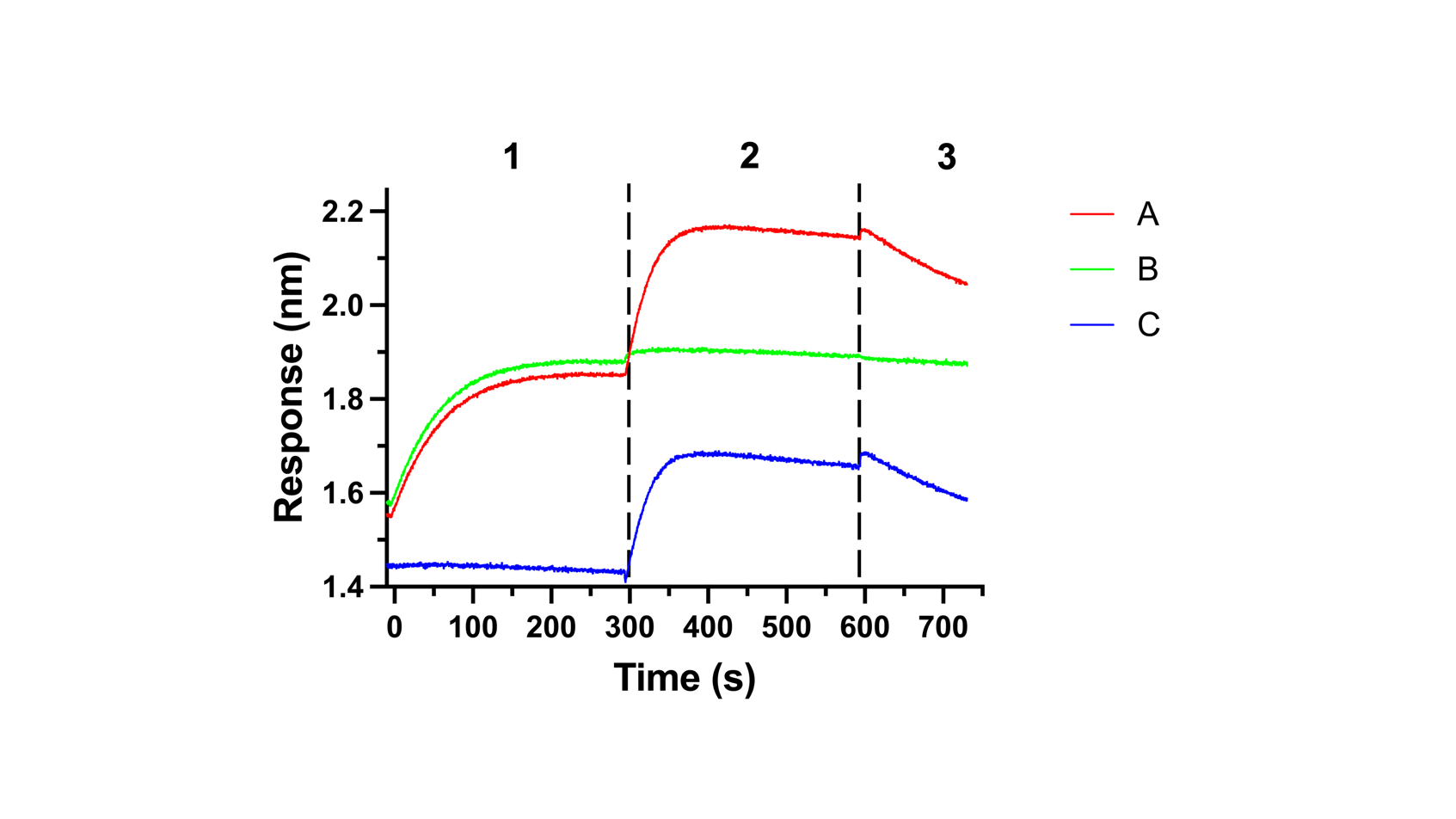
**

**FIGURE S3. Epitope binning experiments confirm the ability of VHH14 and VHH15 to bind to mPlexin-B1 concurrently.** The potential for simultaneous binding of VHH14 and VHH15 was assayed using an Octet RED384 instrument. Biotinylated mouse Plexin-B1 (20-535) was captured using streptavidin (SA) biosensors, and subsequently incubated with VHH-Fcs or assay buffer. Sensor A was incubated with (1) VHH14-Fc and (2) VHH15-Fc; sensor B was incubated with (1) VHH14-Fc and (2) assay buffer. Sensor C was incubated with (1) assay buffer and (2) VHH15-Fc. All VHHs were finally dissociated in assay buffer (3). Simultaneous binding of VHH14-Fc and VHH15-Fc can be observed, suggesting that the VHHs bind to non-overlapping epitopes.

**Figure S4**

**
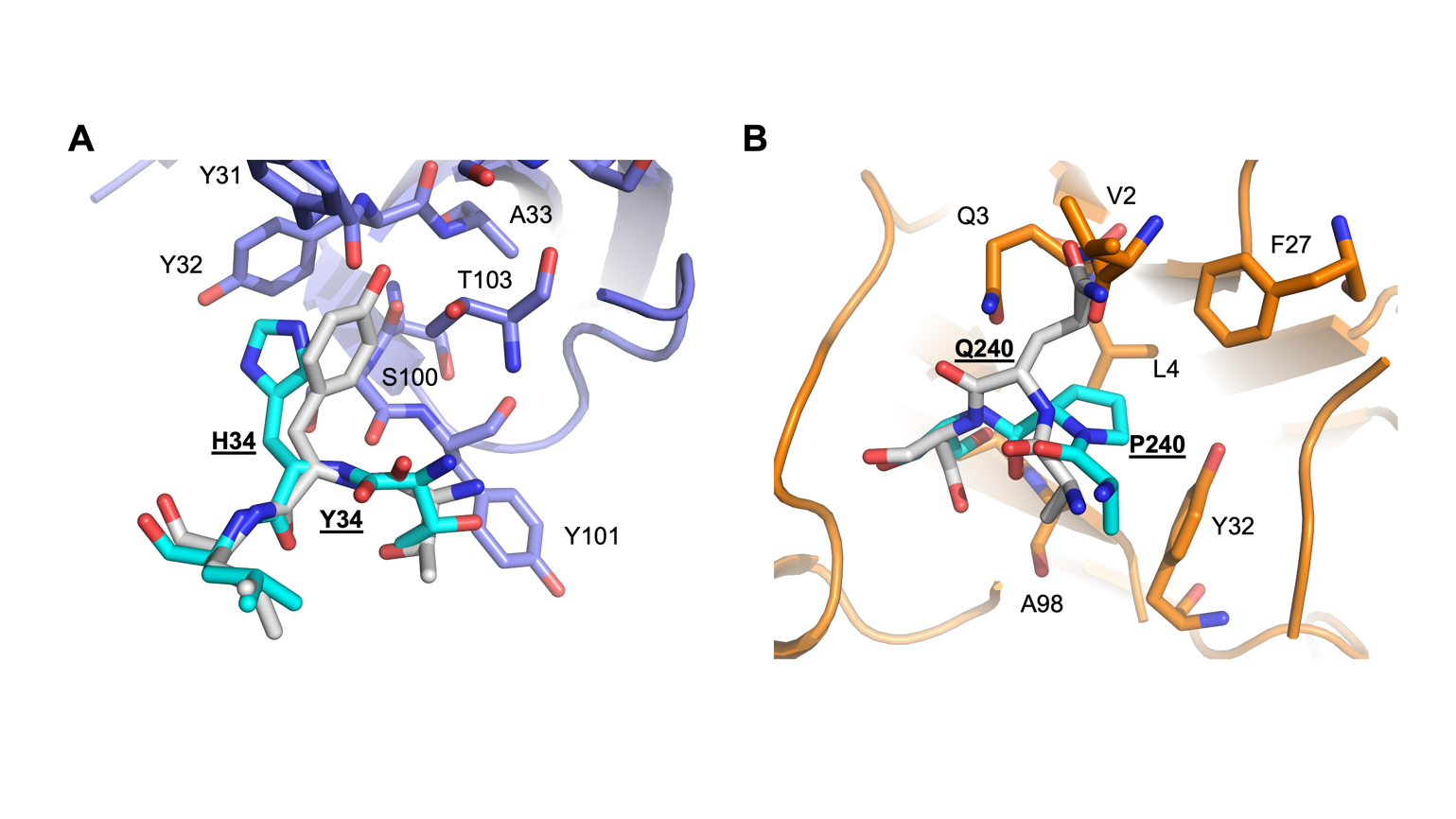
**

**FIGURE S4. Single amino acid changes are found in the binding sites of anti-Plexin-B1 VHHs which contribute to the mouse specificity of the VHHs.** The structural alignment of human Plexin-B1 bound to Sema4D (pdb:3OL2) and mouse Plexin-B1 bound to VHH14 and VHH15 allows the identification of amino acid variations between human and mouse Plexin-B1 which may contribute to the specificity of the VHHs for mouse Plexin-B1 by introducing steric clashes, or removing potential hydrogen bonds. (A) Alignment of mouse Plexin-B1 (cyan) with human Plexin-B1 (grey) allows the identification of the H34/Y34 substitution as being potentially significant to the binding of VHH15 (blue). The residues either side of H34/Y34 from Plexin-B1, and residues from VHH15 surrounding this motif are shown as sticks, with the remainder of VHH15 shown in a cartoon representation. The rest of Plexin-B1 is omitted for clarity. Residue numbers for Plexin-B1 are shown in bold and underlined, and for VHH15 as standard text. (B) Alignment of mouse Plexin-B1 (cyan) with human Plexin-B1 (grey) reveals the P240/Q240 substitution as being potentially significant to the binding of VHH14 (orange). The residues either side of P240/Q240 from Plexin-B1, and residues from VHH14 surrounding this motif are shown as sticks, with the remainder of VHH14 shown in a cartoon representation. The rest of Plexin-B1 is omitted for clarity. Residue numbers for Plexin-B1 are shown in bold and underlined, and for VHH14 as standard text.

**Figure S5**

**
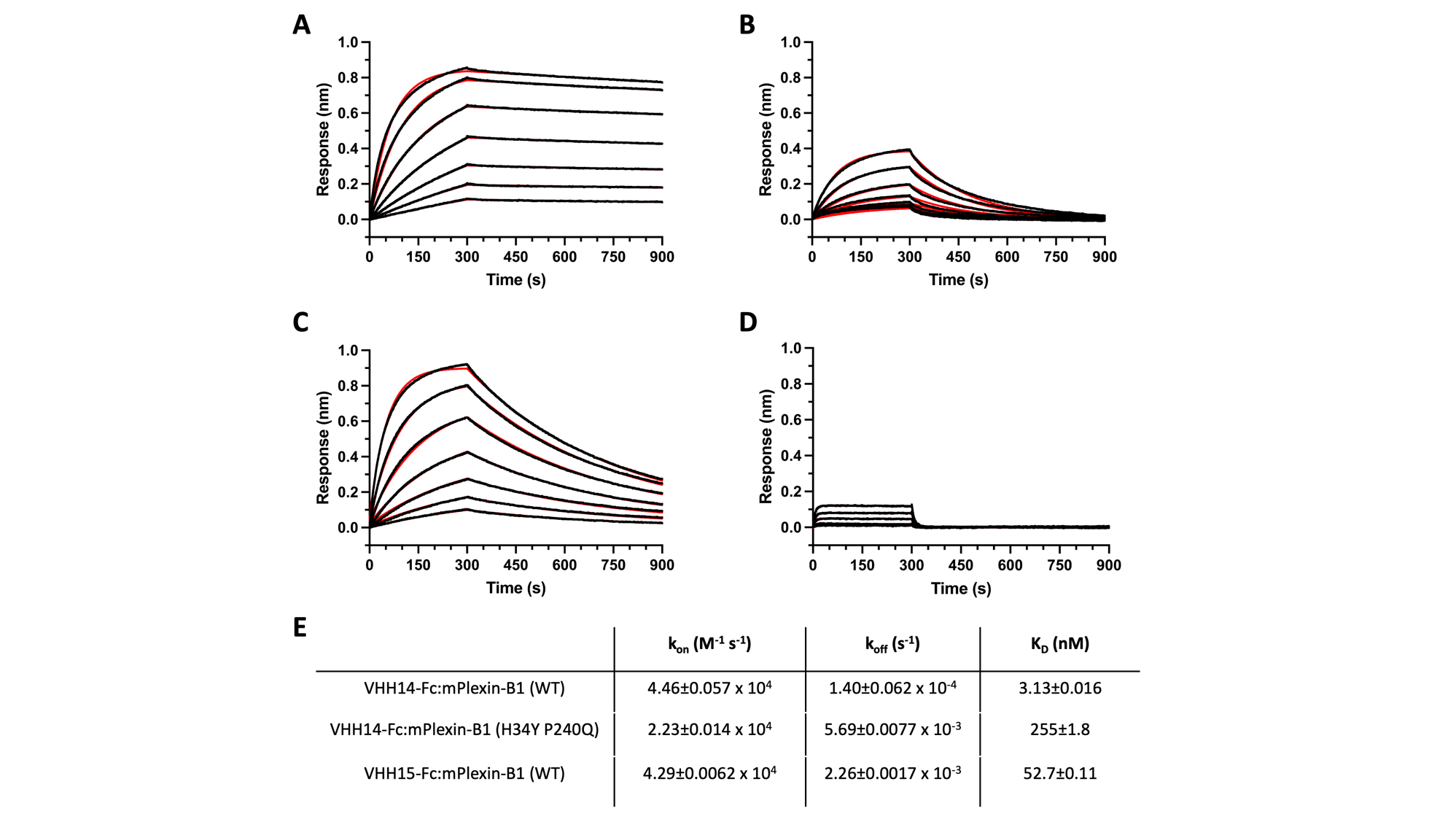
**

**FIGURE S5 Binding of mPlexin-B1 to VHHs is substantially weakened by mouse to human mutations in their respective binding sites.** Comparison of immobilised VHH14-Fc (A, B) or VHH15-Fc (C, D) binding to wild type mPlexin-B1 (20-535) (A, C) or mPlexin-B1 H34Y P240Q (20-535) (B,D). VHH-Fcs were captured using Protein G tips and incubated with 400 nM, 200 nM, 100 nM, 50 nM, 25 nM, 12.5 nM and 6.25nM mouse Plexin-B1 (20-535) WT or H34Y P240Q variant. There is a substantial reduction in the maximum binding response observed for the binding of plexin to the VHHs in the presence of human-equivalent mutations, as well as an increase in the off-rate, showing these changes are mostly responsible for the mouse/human specificity of VHH14 and VHH15. Reference subtracted data is shown in black, and fits for the determination of kinetic parameters are shown in red. (E) Kinetic parameters for the binding of mPlexin-B1 (native and variants) to VHHs obtained by analysis of the association and dissociation curves. Fits for the VHH15-Fc:mPlexin-B1 H34Y P240Q data were unable to be obtained. Data analysis was performed using GraphPad Prism v9.5.1 using a 1:1 association and dissociation model with global analysis of all sensorgrams.

**Figure S6**

**
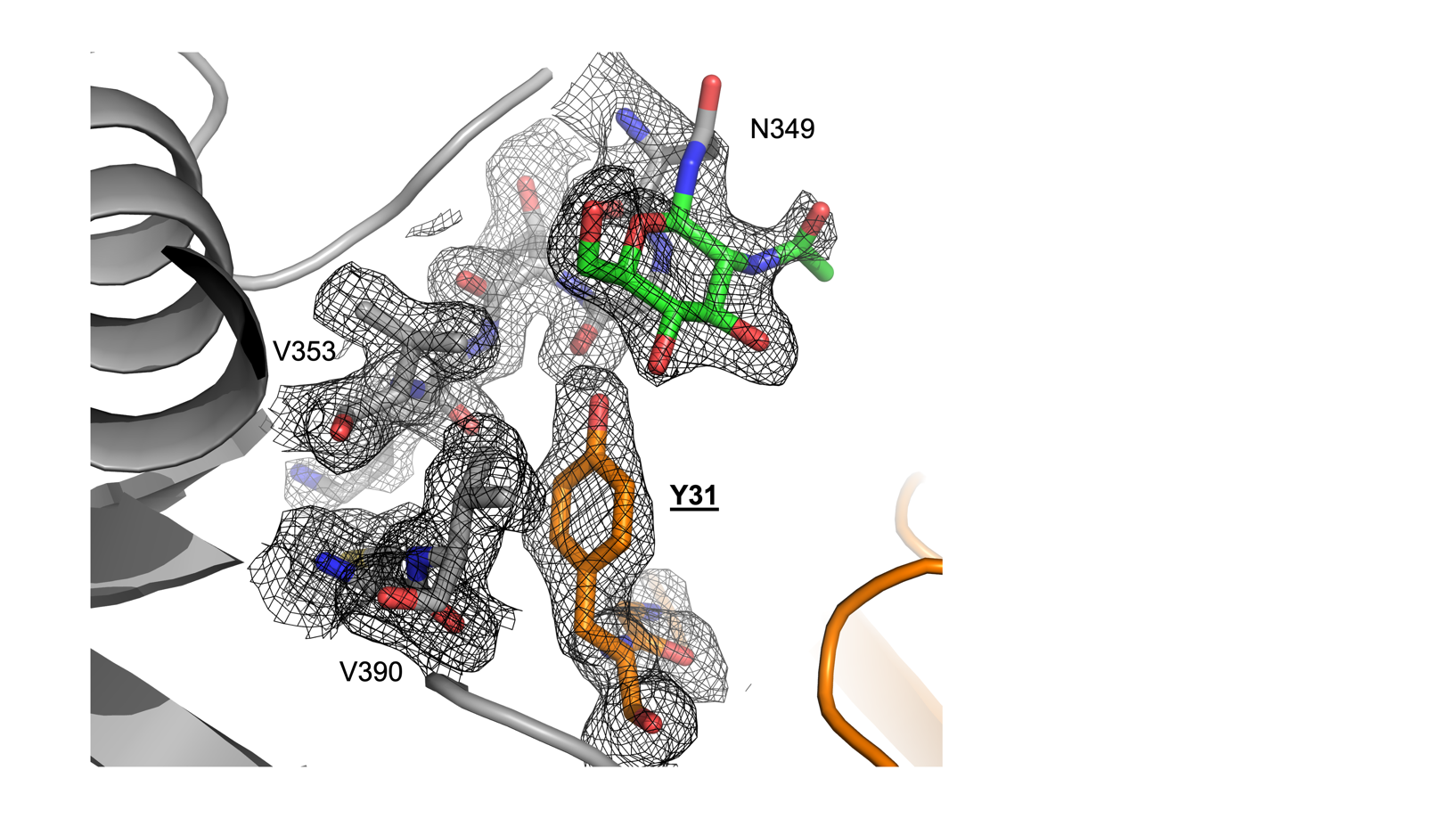
**

**Figure S6. The interaction of VHH14 with the N-linked glycan attached to Asn 349 is well defined in the electron density around the glycan.** The electron density is shown contoured at 1σ, with the key residues around the glycan shown as sticks, with the remainder of mPlexin-B1 and VHH14 shown as a ribbon. VHH14 is coloured orange, mPlexin-B1 is coloured grey and the n-acetylglucosamine is shown in green. Residue numbers from mPlexin-B1 are identified with text, with resides from VHH14 identified with underlined and bold text.

**Figure S7**

**
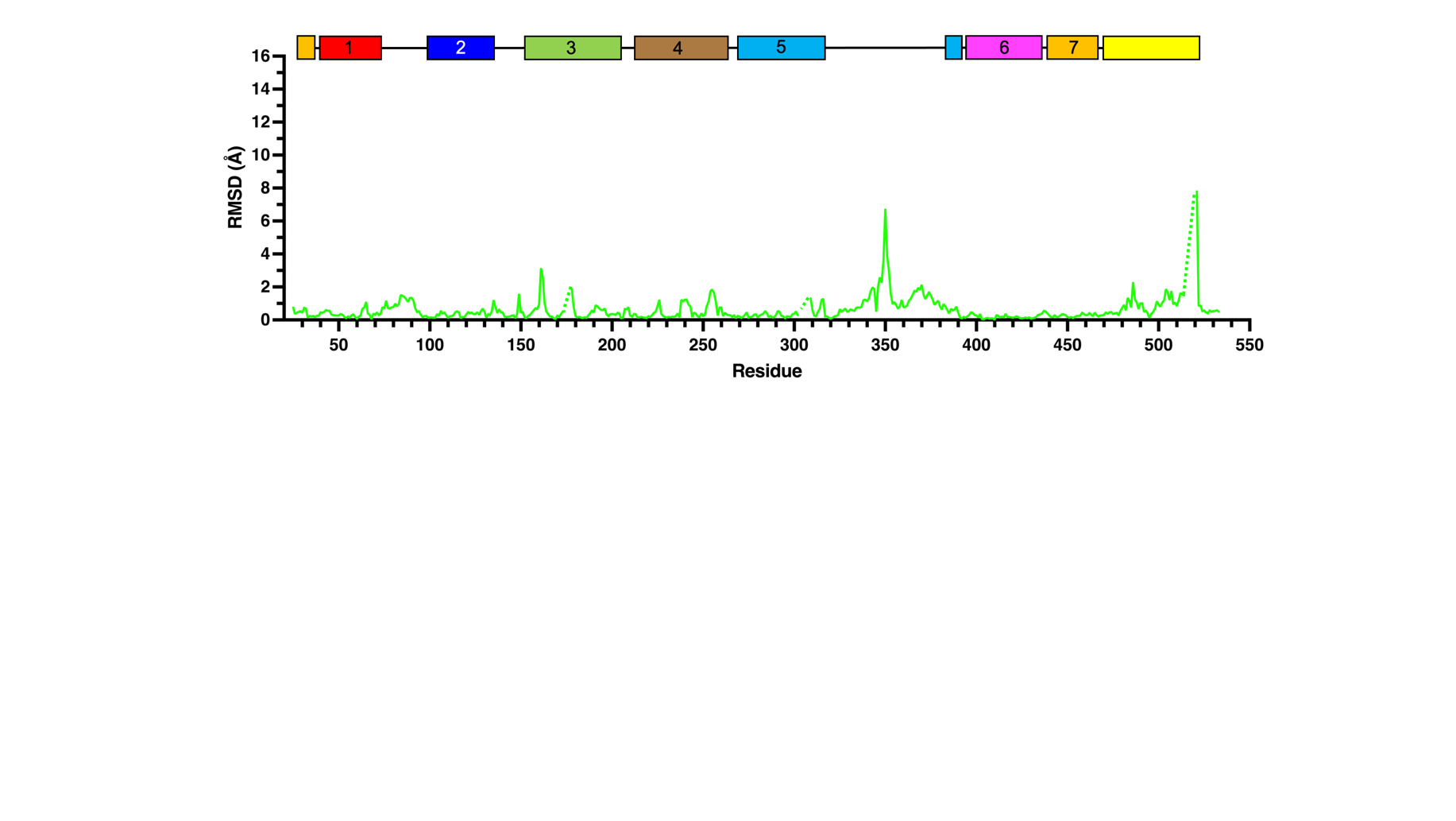
**

**Figure S7 Cα RMSD between chains of human Plexin-B1. Pairwise** Cα RMSDs were calculated between the two chains of apo human Plexin-B1 present in the crystal structure to identify areas of the sema and PSI1 domains which varied in position, in order to act as a reference point for comparisons between apo and Sema4D bound Plexin-B1. Regions for which either chain (A or B) did not contain a modelled residue are indicated by a dotted line. The locations of the seven blades of the sema domain of hPlexin-B1 and the PSI1 domain are indicated above the plot, coloured as Figure 1A.

**Figure S8**


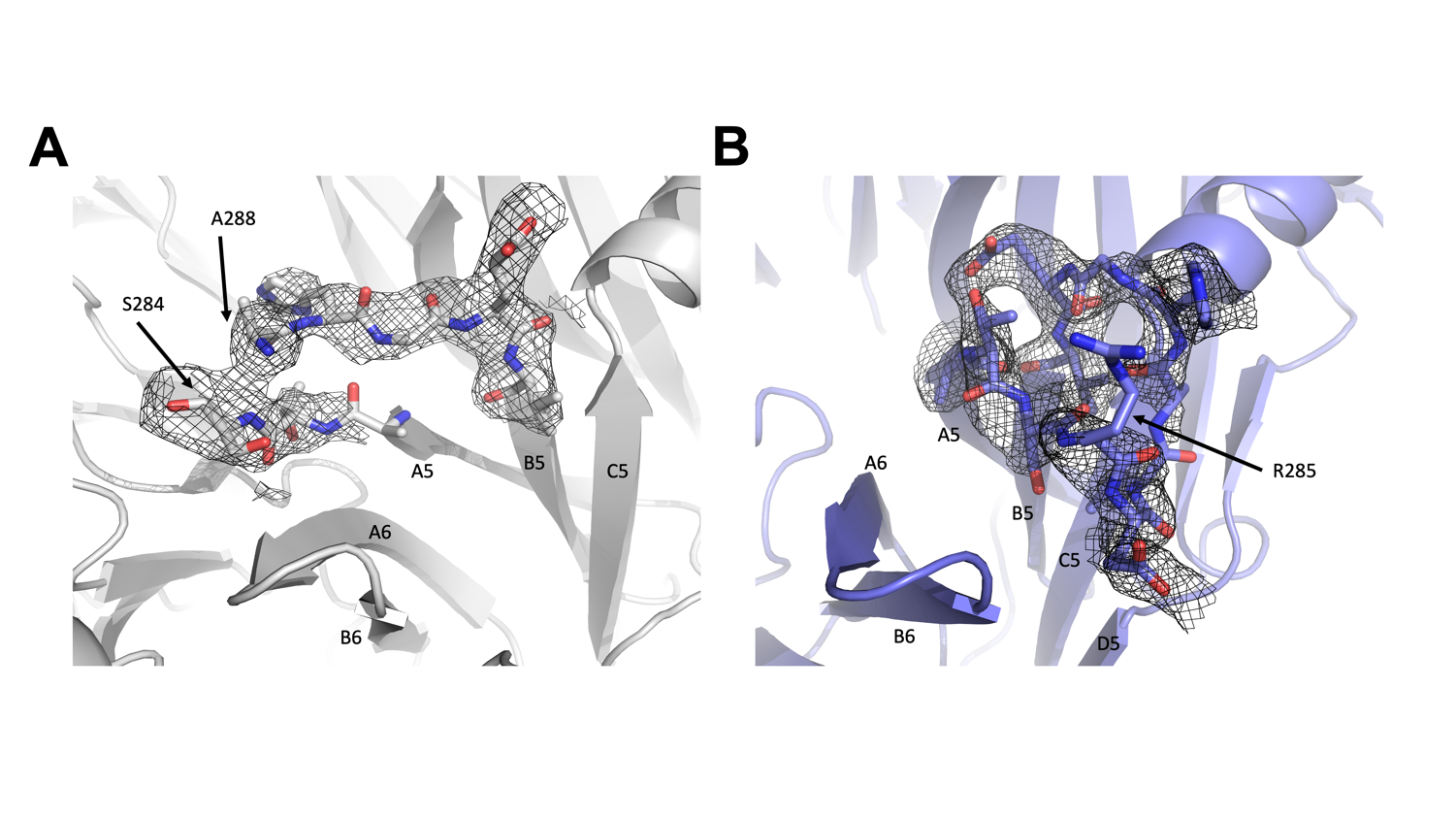


**Figure S8. The A5-B5 loop is well defined in both apo and Sema4D-bound hPlexin-B1 crystal structures.** The electron density around the A5-B5 loop is shown, contoured at 1σ, in the Sema4D-bound (A, pdb:3OL2) and apo (B) forms of hPlexin-B1, with the A5-B5 loop represented as sticks, and the remainder of the sema and PSI1 domains in a ribbon representation. The majority of both loops is fit within the electron density, with the exception of residues 285-287 in Sema4D-bound hPlexin-B1 (A), which are not modelled in the deposited structure, and the side chain of Arg285 in apo hPlexin-B1 (B) for which no density was observed.

**Figure S9**

**
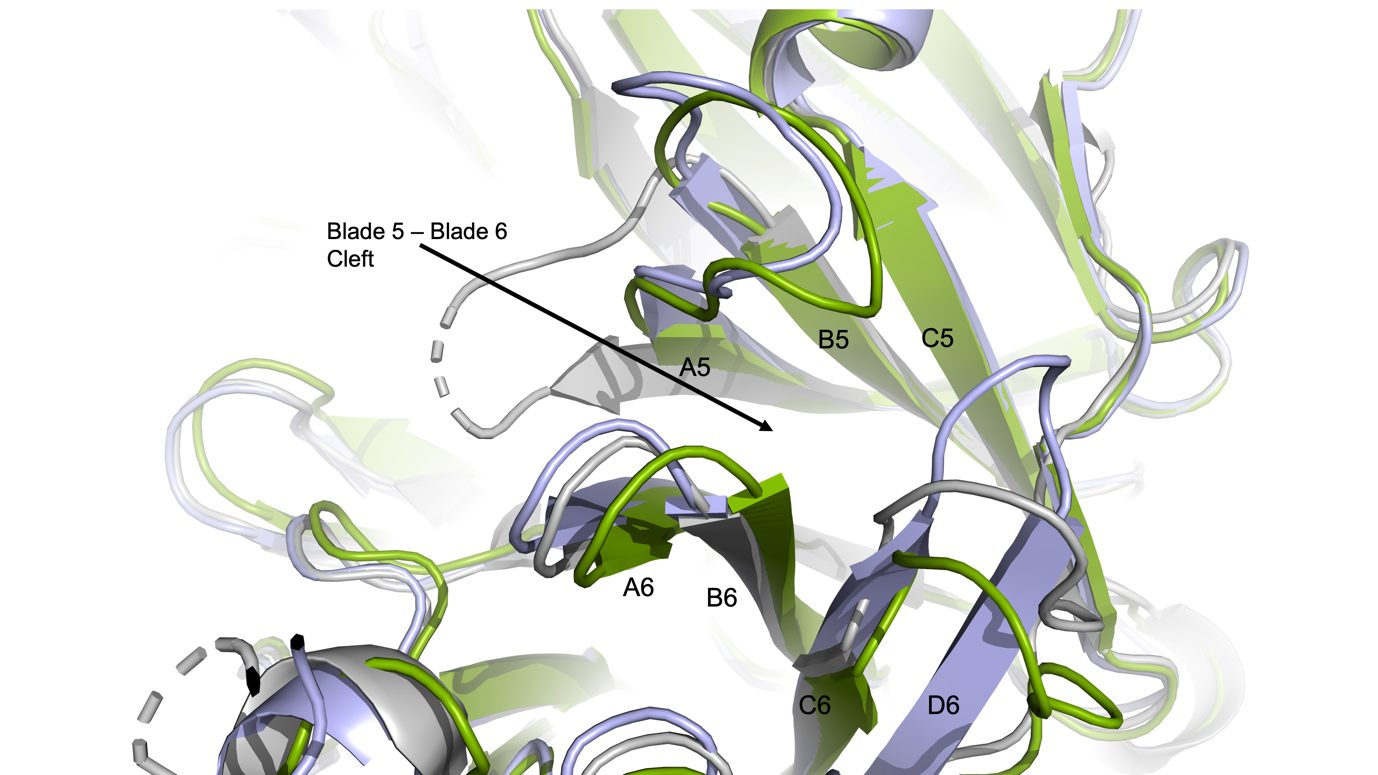
**

**Figure S9 Alphafold and apo hPlexin-B1 structures are similar in the blade 5/6 region of the sema domain.** The Alphafold DB model (O43157-F1 V2) of full length human Plexin-B1 (blue) and the apo structure of hPlexin-B1 (20-535, green) were aligned to the sema domain of the complex of hPlexin-B1 with Sema4D (pdb:3OL2, grey). This illustrates that the Alphafold model adopts a similar closed cleft between the 5^th^ and 6^th^ blades of the sema domain to the apo structure, as opposed to the open cleft seen in the Sema4D-bound structure.

**Figure S10**

**
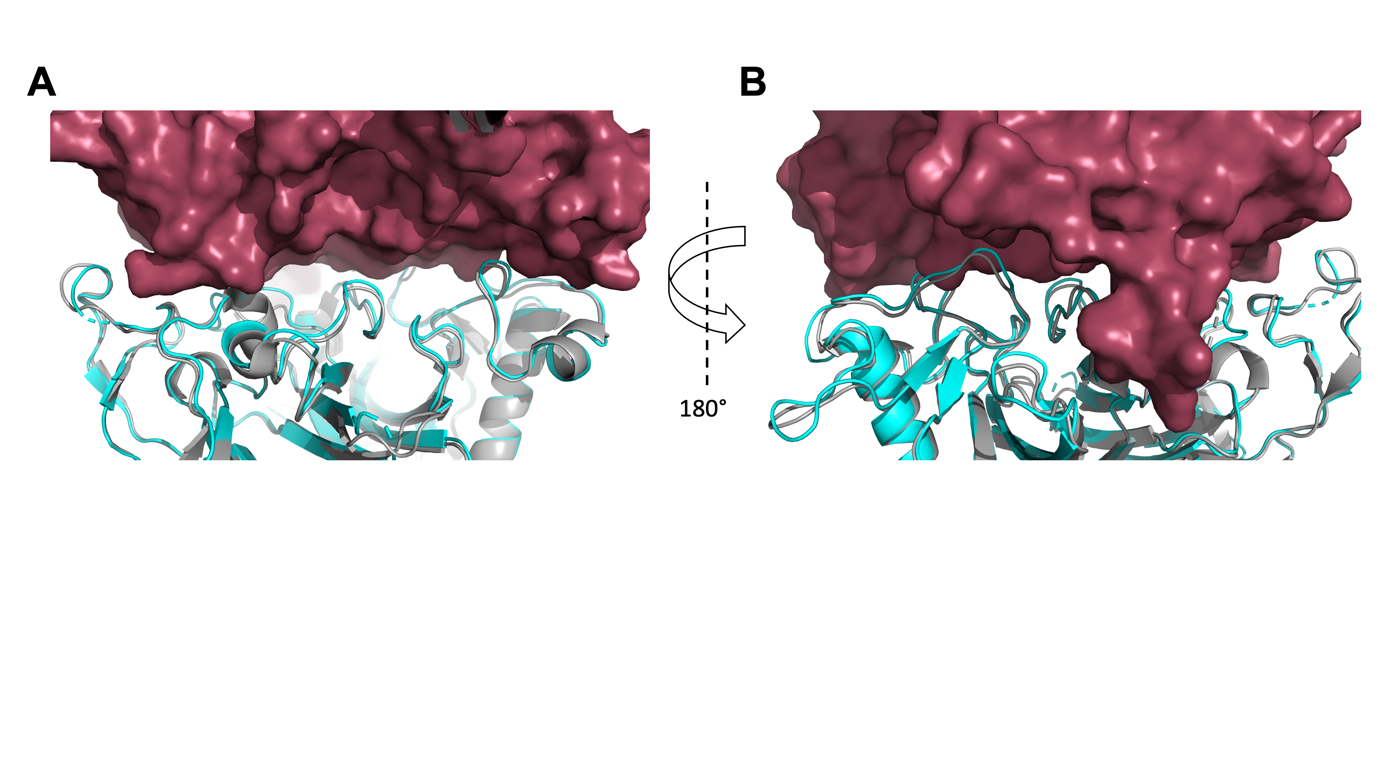
**

**Figure S10 Subtle changes occur in the loops of the sema domain of Plexin-B1 that contact Sema4D on inhibitory VHH binding.** The sema domain of mPlexin-B1 from the VHH14:VHH15:mPlexin-B1 complex (cyan) was aligned to the hPlexin-B1:Sema4D complex (pdb:3OL2, grey). Plexin-B1 domains are shown in a ribbon representation, whilst Sema4D from the hPlexin-B1:Sema4D complex is shown as a surface (red). Although small changes occur in the positioning of the loops in the interface, there is no steric clash that occurs between the mPlexin-B1 sema domain and Sema4D.
